# Anti-Fibrotic Potential of Tomentosenol A, a Constituent of Cerumen from the Australian Native Stingless Bee, *Tetragonula carbonaria*

**DOI:** 10.3390/antiox11081604

**Published:** 2022-08-19

**Authors:** Karina D. Hamilton, Daniel Czajkowski, Nicolas J. Kong, Trong D. Tran, Kirk R. Gustafson, Gary Pauly, Glen M. Boyle, Jacinta L. Simmons, Robert Steadman, Ryan Moseley, Peter R. Brooks, Steven M. Ogbourne, Fraser D. Russell

**Affiliations:** 1 Centre for Bioinnovation, University of the Sunshine Coast, Maroochydore DC, QLD 4558, Australia; 2 School of Health and Behavioural Sciences, University of the Sunshine Coast, Maroochydore DC, QLD 4558, Australia; 3 School of Science, Technology and Engineering, University of the Sunshine Coast, Maroochydore DC, QLD 4558, Australia; 4 Molecular Targets Program, Center for Cancer Research, National Cancer Institute, Frederick, MD 21702-1201, USA; 5 Chemical Biology Laboratory, Center for Cancer Research, National Cancer Institute, Frederick, MD 21702-1201, USA; 6 QIMR Berghofer Medical Research Institute, Locked Bag 2000, PO Royal Brisbane Hospital, Brisbane, QLD 4029, Australia; 7 Wales Kidney Research Unit, School of Medicine, Cardiff Institute of Tissue Engineering and Repair (CITER), Cardiff University, Cardiff CF14 4XN, UK; 8 Regenerative Biology Group, School of Dentistry, Cardiff Institute of Tissue Engineering and Repair (CITER), Cardiff University, Cardiff CF14 4XY, UK

**Keywords:** hypertrophic scars, stingless bee, cerumen, propolis, antioxidant, fibroblasts, keratinocytes, proliferation

## Abstract

Bioactivity-guided fractionation was used to isolate two compounds, tomentosenol A (**1**) and torellianone A (**2**), from a cerumen extract from *Tetragonula carbonaria*. The anti-fibrotic activity of these compounds was examined using human cultured neonatal foreskin fibroblasts (NFF) and immortalised keratinocytes (HaCaTs). Tomentosenol A (**1**), inhibited NFF and HaCaT cell proliferation and prevented NFF and HaCaT scratch wound repopulation at 12.5–25 µM concentrations. These inhibitory effects were associated with reduced cell viability, determined by tetrazolium dye (MTT) and sulforhodamine B (SRB) assays. Compound **1** further inhibited transforming growth factor-β_1_ (TGF-β_1_)-stimulated, NFF-myofibroblast differentiation and soluble collagen production; and was an effective scavenger of the model oxidant, 2,2-diphenyl-1-picrylhydrazyl (DPPH·), with an EC_50_ value of 44.7 ± 3.1 µM. These findings reveal significant anti-fibrotic potential for cerumen-derived tomentosenol A (**1**).

## 1. Introduction

Scar formation and remodelling are final events in the cutaneous wound healing process and provide partial restoration of tissue cohesion and strength following injury. Scar tissue may be contained within the wound region, typical of hypertrophic scars, or exceed the boundary of the wound site, typical of keloid scars [1,2]. Scar tissue is structurally different from normal skin, having elevated collagen levels, increased type I/III collagen ratios and abnormal collagen cross-linking microarchitecture [3]. Elevated extracellular matrix stiffness is characteristic of scar tissue, and this is known to alter keratinocyte migration and proliferation [4,5]. Pain, restricted mobility, and diminished self-image are experienced by some patients who are afflicted by abnormal scarring [6]. While a range of pharmacological and non-pharmacological strategies are used to manage patients with scars, several limitations of these treatment modalities have been reported [3]. New approaches aimed at curtailing scar formation are warranted, with growing interest in identifying novel therapeutics from natural products [7].

The nests of *Apis mellifera* honeybees are built primarily from wax and their hives are sealed by propolis resin. In contrast, the nests of stingless bees belonging to the tribe Meliponini are made primarily from a propolis-based substance called cerumen, because they do not utilize hexagonal beeswax combs [8]. Cerumen supports nest structures and protects bees against predators and diseases [9]. Propolis has been reported to initiate collagen remodelling in granulation tissue in a manner that is suggestive of anti-scarring and anti-fibrotic properties [10]. Although stingless bee cerumen and honeybee propolis are similar resinous materials, stingless bee cerumen has lower structural diversity (i.e., a smaller number of molecules) but higher scaffold diversity (number of core structures comprising rings and linkers) [11]. *Tetragonula carbonaria* is a social stingless bee species that is native to Australia [12] and represents a novel source of cerumen that has not been widely studied. Previous nuclear magnetic resonance analyses revealed the presence of tetragocarbone A and tetragocarbone B in cerumen of *T. carbonaria* [13], but their bioactivities were not investigated. Other chemical constituents reported from cerumen of *T. carbonaria* include (2*S*)-cryptostrobin, (2*S*)-stroboponin, (2*S*)-cryptostrobin 7-methyl ether, (2*S*)-pinostrobin, (2*S*)-pinocembrin, (2*S*)-desmethoxymatteucinol and abietic acid [14]. Using crude cerumen extracts, our group has previously reported vasorelaxant, anti-bacterial and antioxidant activities of cerumen from *T. carbonaria* [15,16,17,18]. In this study, we isolated two compounds from the bioactive cerumen extract, neither of which have been previously reported as constituents of cerumen from *T. carbonaria*. The compounds, identified as tomentosenol A (**1**) and torellianone A (**2**), were investigated for cell-based activities that are implicated in the prevention of scarring and fibrosis.

## 2. Materials and Methods

### 2.1. General Experimental Procedures

Optical rotations ([α]_D_), UV spectra, ECD spectra and NMR spectra were acquired as previously described [19]. Spectra of ^1^H and ^13^C were referenced to the residual deuterated solvent peaks at *δ*_H_ 2.50 and *δ*_C_ 39.5 (DMSO-*d*_6_), and at *δ*_H_ 7.26 and *δ*_C_ 77.2 (CDCl_3_). Heteronuclear multiple bond correlation (HMBC) experiments were optimized for ^n^*J*_CH_ = 8.3 Hz. HRESIMS data were acquired on an Agilent 6520 Accurate Mass Q-TOF spectrometer. High performance liquid chromatography (HPLC) purifications were performed using a PerkinElmer Series 200 HPLC pump and autosampler, with a Flexar photodiode array detector and the associated Chromera version 3.4.1 software; with delivery module, detector, software and extraction and chromatography solvents and as described previously [19].

### 2.2. Cerumen Material

Cerumen was collected from 40 hives of *T. carbonaria* in the metropolitan Brisbane region of South-East Queensland, Australia by Professor Tim Heard.

### 2.3. Extraction and Isolation

A methanol extract of cerumen from *T. carbonaria* was prepared as previously described [17]. Dried methanol-water extract (100 mg) was reconstituted in 1 mL of 70% mobile phase (MP) A/30% MPB (MPA: 95% H_2_O, 5% CH_3_CN; MPB: 10% H_2_O, 90% CH_3_CN). The entire volume was manually injected onto a Kinetex C_18_ column (4.0 μm, 100 mm × 22 mm) and fractionated at a flow rate of 4.0 mL/min with a contiguous elution series consisting of 70% MPA, 30% MPB for 5 min, a linear gradient from 70% MPA/30% MPB to 47% MPA/53% MPB over 40 min, 47% MPA/53% MPB for 20 min, a linear gradient from 47% MPA/53% MPB to 100% MPB over 80 min, and 100% MPB for 30 min. Eleven fractions (16 min per fraction) were collected.

Bioactive fraction 9 [18] was dissolved in hot MeOH. The solution was slowly cooled to obtain Compound **1** (3.8 mg) as colourless needles. The supernatant was further purified on a C_18_ HPLC column (5 µm, 250 mm × 10 mm) at a flow rate of 4.0 mL/min for 30 min, with a linear gradient from 10% CH_3_CN/90% H_2_O (0.1% trifluoroacetic acid [TFA]) to 50% CH_3_CN/50% H_2_O (0.1% TFA) over 5 min, 50% CH_3_CN/50% H_2_O (0.1% TFA) to 100% CH_3_CN (0.1% TFA) over 20 min, and 100% CH_3_CN (0.1% TFA) for 5 min to yield Compound **2** (1.9 mg, t_R_ = 20.1 min).

Tomentosenol A (**1**): colourless rectangular plate-like crystal; [α]^23^_D_ −12.5 (*c* 0.04, MeOH); UV (MeOH) *λ*_max_ (log *ε*) 290 (3.61) nm; ^1^H and ^13^C NMR data, Table S1 (Supporting Information); (+) HRESIMS *m/z* 387.2898 [M + H]^+^ (calculated for C_25_H_39_O_3_^+^, 387.2894, Δ1.0 ppm).

Torellianone A (**2**): white amorphous powder; [α]^23^_D_ +115.0 (*c* 0.05, MeOH); UV (MeOH) *λ*_max_ (log *ε*) 225 (4.11), 263 (3.60), 307 (3.85) and 355 (3.28) nm; ECD (*c* 532 × 10^−6^ M, MeOH) *λ*_max_ (Δ*ε*) 226 (+12.33), 263 (−8.10) and 305 (+5.29) nm; ^1^H and ^13^C NMR data, Table S2 (Supporting Information); (+) HRESIMS *m/z* 523.2126 [M + H]^+^ (calculated for C_33_H_31_O_6_^+^, 523.2115, Δ2.1 ppm).

### 2.4. Crystallographic Data for Tomentosenol A (**1**) 

Tomentosenol A (**1**): C_25_H_38_O_3_, MW 386.55, *T* 100 K, approximate dimensions 0.030 mm × 0.300 mm × 0.450 mm, was used for the X-ray crystallographic analysis. The X-ray intensity data were measured on a Bruker D8 QUEST system equipped with a multilayer mirror monochromator and a Cu Kα microfocus sealed tube (*λ* = 1.54178 Å). A total of 8087 frames were collected. The total exposure time was 45.18 h. The frames were integrated with the Bruker SAINT software package using a narrow-frame algorithm. The integration of the data using a monoclinic unit cell yielded a total of 77,026 reflections to a maximum θ angle of 72.19° (0.81 Å resolution), of which 8930 were independent (average redundancy 8.626, completeness = 99.5%, R_int_ = 4.05%, R_sig_ = 2.21%) and 8837 (98.96%) were greater than 2*σ* (F^2^). The final cell constants of a = 11.3184 (19) Å, b = 15.3858 (15) Å, c = 14.184 (2) Å, β = 111.833 (9)°, volume = 2292.9 (6) Å^3^, were based upon the refinement of the XYZ-centroids of 138 reflections above 20*σ* (I) with 0.989° < 2θ < 119.0°. Data were corrected for absorption effects using the Multi-Scan method (SADABS). The ratio of minimum to maximum apparent transmission was 0.740. The calculated minimum and maximum transmission coefficients (based on crystal size) were 0.7880 and 0.9840.

### 2.5. Structure Determination by X-ray Diffraction Analysis

The structure was solved and refined using the Bruker SHELXTL Software Package, using the space group *P*12_1_1, with Z = 4 for the formula unit, C_25_H_38_O_3_. The final anisotropic full-matrix least-squares refinement on F^2^ with 527 variables converged at R1 = 2.97% for the observed data and wR2 = 7.96% for all data. The goodness-of-fit was 1.042. The largest peak in the final difference electron density synthesis was 0.286 e^−^/Å^3^ and the largest hole was –0.165 e^−^/Å^3^ with an RMS deviation of 0.031 e^−^/Å^3^. Based on the final model, the calculated density was 1.120 g cm^3^ and F (000), 848 e^−^.

### 2.6. Computational Details

Computational details pertaining to **2** were as described previously [19], with minor modifications: Internal relative energy of conformers was 10 kcal/mol. Optimized conformers were then subjected to TDDFT calculations in MeOH on Gaussian 09 using the CAM-B3LYP functional and the SV basis set.

### 2.7. Biological Assays

Cell Culture and Reagents. This study used neonatal foreskin fibroblasts (NFF) and a spontaneously transformed immortal human epidermal keratinocyte cell line (HaCaT) [20]. The HaCaT cells were obtained from Dr. Nicholas Saunders, University of Queensland, Australia. Both cell types were maintained in Roswell Park Memorial Institute-1640 (RPMI) media supplemented with 10% foetal calf serum (FCS), 1% L-glutamine (2 mM), 100 U/mL penicillin G and 100 µg/mL streptomycin (10% RPMI).

Cell Survival Assays. Cell survival was measured in the HaCaT and NFF cultures (1.5 × 10^4^ cells per well) exposed to tomentosenol A (**1**), prepared as a stock solution in 100% DMSO and diluted with RPMI media. A control comprised an equivalent percentage of DMSO (0.05%) to that present in the highest concentration of (**1**). Cell survival was assessed in 24-well plates using an MTT (3-(4,5-dimethylthiazol-2-yl)-2,5-diphenyl tetrazolium bromide) dye-reduction assay and a sulforhodamine B (SRB) assay. For the MTT assay, cells were maintained in 10% RPMI for 24 h in a 5% CO_2_ incubator at 37 °C. Media was exchanged for serum-free media and cells were incubated for 24 h before switching to RPMI containing 1% FCS (1% RPMI) and 0.75–25 μM cerumen compounds or an equivalent volume of DMSO. Cells were incubated for 24 h before replacing media with 200 µL of MTT extraction buffer (10% DMSO in 0.5 M N,N-dimethylformamide; pH 4.7). After a 4-h incubation at 37 °C, solubilisation solution (10% sodium dodecyl sulfate solution prepared in milliQ water and containing 0.01 M HCl) was added and samples were incubated overnight 37 °C. The next day, the cell solution was mixed using a pipette, and single 100 μL aliquots were added to a 96-well plate and read at 570 nm (*n* = 8). For the SRB assay, NFF cells were seeded into 24-well plates (Thermo Fisher Scientific, Scoresby, Australia) at a density of 1.5 × 10^4^ cells per well and incubated for 24 h at 37 °C and 5% CO_2_ (250 µL/well in 10% RPMI). After incubation, the media was removed and replaced with serum-free media. Following a further 24-h incubation, the media replaced with 250 µL of 1% RPMI, with or without tomentosenol A (0.75 µM to 25 µM). A solvent control (1% RPMI containing an equivalent volume of DMSO) was included. Cells were incubated for 24 h before adding 100 µL of cold 10% (*w*/*v*) trichloroacetic acid (TCA). Cells were incubated at 4 °C for 1 h to promote cell fixation to the bottom of the well. Cells were gently washed with tap water and aspirated four times. Plates were allowed to air-dry at room temperature (20 °C) before adding 200 µL of 0.057% (*w*/*v*) sulforhodamine B (SRB) solution to each well. Plates were incubated at 20 °C for 30 min before rinsing with 1% (*v*/*v*) acetic acid to remove unbound dye. Plates were air-dried at 20 °C. Next, 200 µL of 10 mM Tris base solution (pH 10.5) was added to each well and plates were placed on a shaker for 5 min to facilitate the solubilisation of protein-bound dye. Aliquots (100 µL) from each well were transferred into a 96-well plate and absorbance was read at 510 nm.

Cell Proliferation Assay. The effect of Compound **1** on cell proliferation was investigated using the IncuCyte Zoom Live-Cell Analysis System (Essen Bioscience, Ann Arbor, Michigan; *n* = 3). NFF and HaCaT cells were seeded into 96-well Corning plates (Thermo Fisher Scientific, Australia) at a density of 2.5 × 10^3^ cells per well in 10% RPMI to achieve 10–30% confluence. Compound **1** was added to each well in triplicate (0.75–50 μM), or DMSO at an equivalent concentration to that of 50 µM Compound **1** (*n* = 3). Plates were incubated in the IncuCyte Zoom and cell proliferation was recorded at 2-h intervals over 72 h. Data were analysed using IncuCyte Zoom 2016A software.

Scratch Wound Repopulation Assay. The effect of Compound **1** on cell migration was investigated using an IncuCyte Zoom Live-Cell Analysis System. NFF and HaCaT cells were seeded into 96-well ImageLock^TM^ plates (Essen Bioscience) in 10% RPMI at a density of 1.25 × 10^4^ cells per well. After cell confluence was achieved, a single scratch through the cell monolayers was made using the Essen Bioscience WoundMaker^TM^ (~1200 µm in width). Cells were washed twice in PBS before adding Compound **1** (0.75–50 µM) or DMSO at an equivalent concentration to that of 50 µM Compound **1** (*n* = 3). Cell migration into the wound region was measured at 2-h intervals for 72 h using the IncuCyte Zoom. Data were analysed using IncuCyte Zoom 2016A software.

Microscopy and Image Analysis. Myofibroblast differentiation in response to Compound **1** was examined using confocal microscopy. The NFF cells were seeded into 8-well chamber slides (Starstedt Australia) at a density of 8.4 × 10^3^ cells per well. Cells were maintained in 10% RPMI (200 µL per well) for 48 h at 37 °C and 5% CO_2_, then in serum-free media for a further 48 h. The media was replaced with 1% RPMI containing Compound **1** (3.1–25 µM), or an equivalent volume of DMSO, and 10 ng/mL recombinant TGF-β_1_, (Sigma Aldrich, Ltd., St. Louis, MO, USA) then incubated for 72 h. Cells incubated without Compound **1** or TGF-β_1_ served as controls. Cells were fixed with 4% paraformaldehyde in PBS (200 µL; 10 min), rinsed three times in PBS, treated with 0.1% Triton X-100 in PBS (150 µL, 5 min), rinsed three times in PBS and then blocked with 1% BSA in PBS for 1 h. The cells were rinsed three times with 0.1% BSA in PBS and then incubated overnight at 4 °C with monoclonal mouse anti-human α-SMA clone 1A4 (1:100–1:200 in 0.1% BSA/PBS; 150 µL per well; catalogue number A2547; Sigma Aldrich, Ltd.). The cells were washed with 0.1% BSA in PBS (3 × 1 min; 3 × 5 min), then treated with Alexa Fluor 488 (H + L) secondary antibody (goat anti-mouse IgG; 1:1000 in 0.1% BSA in PBS; 150 µL per well; catalogue number 4408S, Cell Signalling Technology, Danvers, MA, USA) and incubated in the dark for 1 h at room temperature. The cells were washed with 0.1% BSA in PBS (3 × 1 min; 3 × 5 min), before and after incubation with a nuclear stain for 30 min at room temperature (DAPI, 2 µg/mL in 0.1% BSA in PBS). The chambers were removed and slides were mounted with glycerol (*n* = 2). Photomicrographs were captured using a Nikon Eclipse Ti inverted confocal microscope with a Nikon DS-Qi1Mc 1.5-MP monochrome camera attachment.

5-Lipoxygenase enzyme activity assay. The 5-lipoxygenase activity assay was carried out using the protocol described in Massaro et al., 2011 [15], with the following modifications. Samples were incubated with 450 units of 5-lipoxygenase. The sample containing tomentosenol A was compared to an equivalent volume of DMSO prepared in methanol. Absorbance (590 nm) was measured in a 96 well plate using an EnSpire Multimode plate reader (PerkinElmer).

2,2-Diphenyl-1-picrylhydrazyl (DPPH·) Free Radical Scavenging Assay. Free radical scavenging activity of Compound **1** was measured using a cell-free DPPH· activity assay. For potency determination, Compound **1** (0.5–259 µM; *n* = 3), or equivalent volumes of DMSO (*n* = 3) were incubated with 100 µM DPPH· in methanol for 30 min at 21 °C. Absorbance was read at 518 nm and DPPH· scavenging activity was calculated using Equation (1):

DPPH· scavenging activity (%) = 100 − [(Absorbance of sample ÷ Absorbance of negative control) × 100](1)

A standard curve for gallic acid (2.4–58.8 µM) was constructed. Maximal free radical scavenging activity and EC_50_ values were determined for Compound **1** and gallic acid. The radical scavenging capacity was determined by incubating Compound **1** (0.5–259 μM) with 988 µM DPPH· in ethanol for 30 min at 21 °C, and measuring absorbance at 518 nm at 1-min intervals for 30 min. The slope of the plot of ln (% DPPH· remaining) against time provided the first order rate constant, *K*. The slope of the plot of *K* against the concentration of Compound **1** provided the radical scavenging capacity.

Soluble collagen production. Soluble collagen concentration was determined using a soluble collagen assay kit (Abcam, Cat#Ab241015). The collagen standards were prepared by adding 65–80 μL collagen assay buffer to 0–15 μL of 0.2 mg/mL collagen standard, into a 96 well plate. Collagen assay buffer (60 μL) was added to 20 μL sample, into other wells. Collagenase enzyme mixture (20 μL of a 1:10 dilution of the manufacturer’s stock solution), or 20 μL of collagen assay buffer (background control) was added to the wells. The plate was incubated at 37 °C for 60 min. Detection reaction solution (75 μL) was added to all wells and the plate was incubated at 37 °C for 5 min in the dark. Developer working solution (25 μL) was added to all wells and the plate was incubated at 37 °C for a further 15 min in the dark, with occasional rocking. Fluorescence was measured at excitation/emission wavelengths of 376/468 nm in an EnSpire Multimode plate reader (PerkinElmer, Waltham, MA, USA).

## 3. Results and Discussion

**Figure 1:**
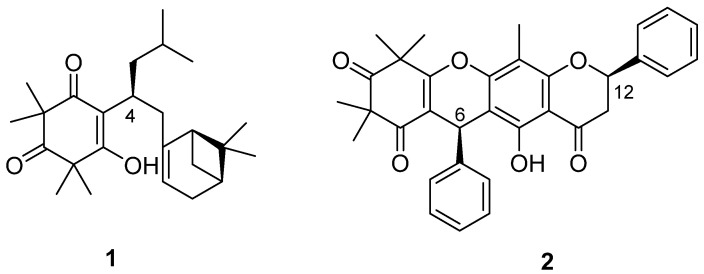
Structures of tomentosenol A (Compound **1**; (**1**)) and torellianone A (Compound **2**; (**2**)).

Compound **1** (Figure 1) was obtained as colourless crystalline needles. Its (+) high resolution electrospray ionization mass spectrometry (HRESIMS) spectrum showed a protonated ion at *m/z* 387.2898, corresponding to a molecular formula of C_25_H_38_O_3_ with seven degrees of unsaturation. Compound **1** displayed two complete sets of proton signals with a ratio of 1:2 in CDCl_3_, but only one set of proton resonances was observed in MeOH-*d_4_*.

**Figure 2:**
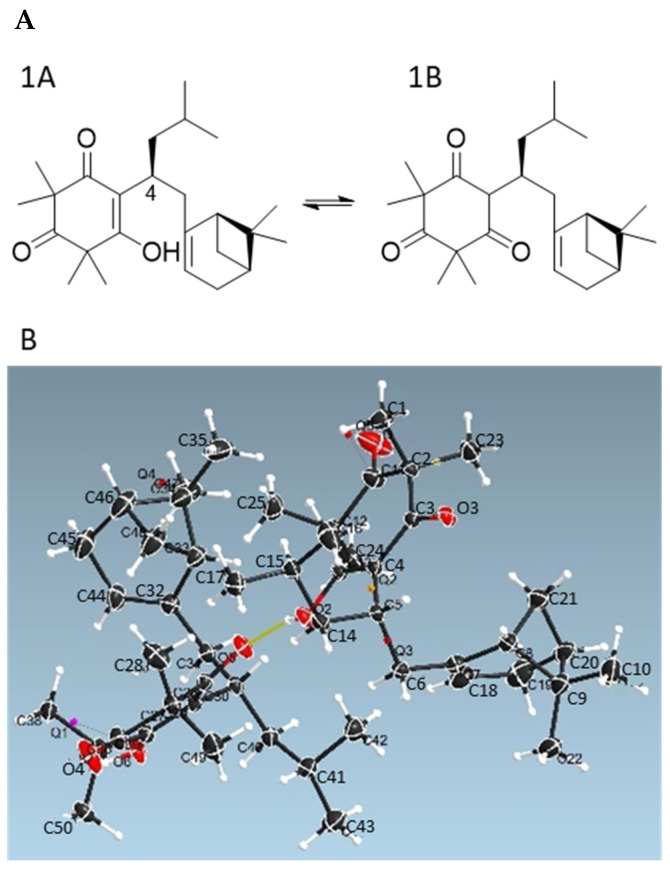
(**A**) Two tautomeric forms **1A** and **1B** of **1**. (**B**) Oak Ridge Thermal Ellipsoid Plot (ORTEP) drawing of **1A**.

Detailed NMR analysis indicated that Compound **1** consisted of two tautomeric forms (**1A**:**1B** = 1:2) in CDCl_3_, while only a single tautomer **1A** was observed in MeOH-*d_4_* (Figure 2A and Table S1, Supporting Information). The NMR data of Compound **1** in CDCl_3_ were consistent with those reported for tomentosenol A, which was isolated from the leaves of *Rhodomyrtus tomentose*, a shrub that is native to South and Southeast Asia [21]. To confirm the 3D structure of Compound **1**, X-ray diffraction analysis of an isolated crystal was acquired using Cu Kα radiation (Figure 2B), allowing an unambiguous assignment of the structure and absolute configuration as (4*S*,1′*R*,5′*S*)-tomentosenol A (**1**).

**Figure 3:**
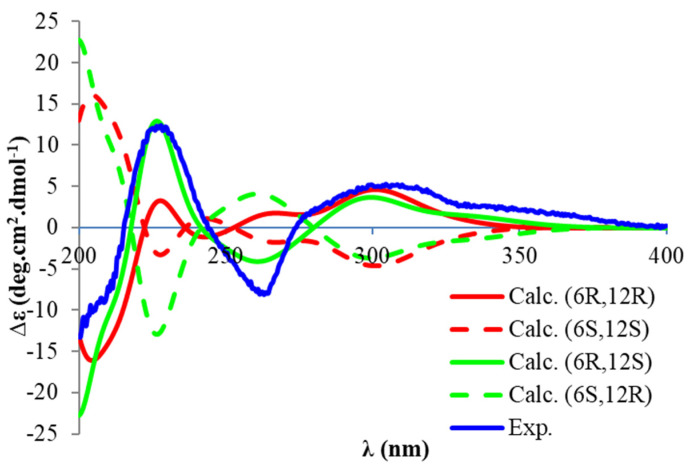
Experimental electronic circular dichroism (ECD) spectrum of **2** and calculated ECD spectra of (6*R*,12*R*) −**2**, (6*S*,12*S*) −**2**, (6*R*,12*S*)-**2** and (6*S*,12*R*) −**2**.

Compound **2** (Figure 1) was purified as a white amorphous powder, with a molecular formula of C_33_H_30_O_6_ deduced from a protonated (+) HRESIMS ion at *m/z* 523.2126. The ^1^H NMR spectrum of Compound **2** was similar to that of torellianone A (Figure S20, Supporting Information), which was identified from the flowers of *Corymbia torelliana* [22]. Detailed 1D and 2D NMR analyses confirmed the structure of Compound **2** as torellianone A (Table S1, Supporting Information). It should be noted that torellianone A was previously isolated as an equimolar mixture with its diastereomer, torellianone B [22]. We isolated torellianone A as a pure compound showing two positive Cotton Effects (CEs) at 226 nm and 305 nm, and one negative CE at 263 nm. Its absolute configuration was determined using quantum chemical electronic circular dichroism (ECD) calculations. Four 3D structural possibilities of **2** were submitted to time-dependent density functional theory (TD-DFT) calculations. The ECD spectrum of the (6*R*,12*S*)-stereoisomer was consistent with the experimental ECD (Figure 3). Therefore, the absolute configuration of Compound **2** was established as (6*R*,12*S*)-torellianone A.

**Figure 4:**
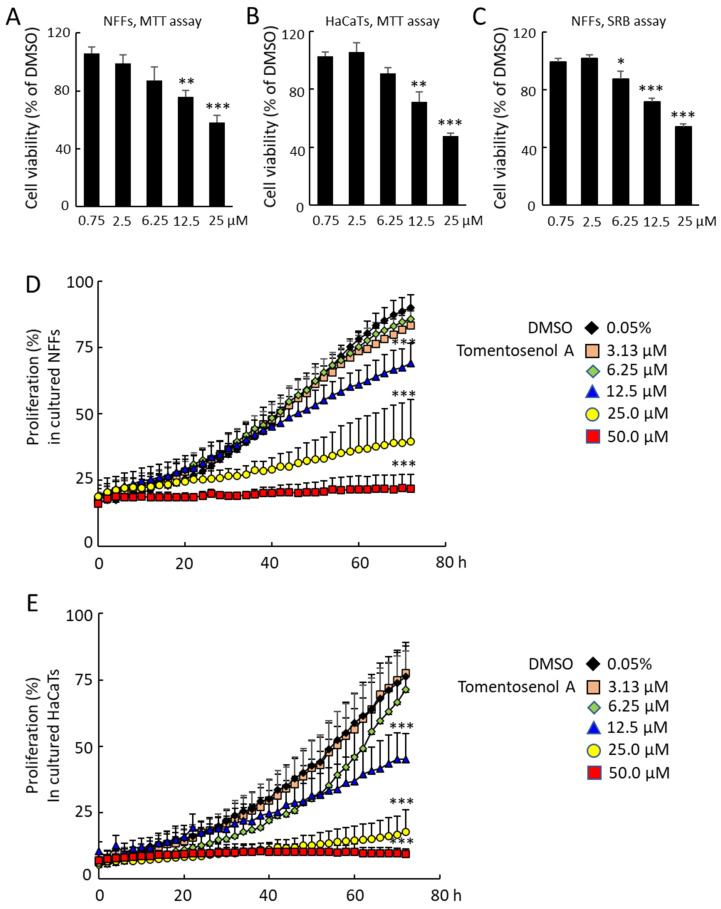
Effects of Compound **1** on neonatal human foreskin fibroblast (NFF) and immortalised keratinocyte (HaCaT) cell viability ((**A**,**B**), MTT assay; (**C**), sulforhodamine B assay), and proliferation. The NFF (**A**,**C**), and HaCaT cells (**B**) were incubated with Compound **1** for 24 h. Cell viability was determined following 4 h incubation with MTT (3-(4,5-dimethylthiazol-2-yl)-2,5-diphenyltetrazolium bromide), or 20 min incubation with sulforhodamine B. Suppression of NFF (**D**), and HaCaT cell (**E**) proliferation during 72-h treatment with Compound **1**. Data are mean ± SEM for *n* = 8 (**A**–**C**) or *n* = 3 (**D**,**E**) independent experiments. * *p* < 0.05, **, *p* < 0.01, ***, *p* < 0.001. Data were analysed using a Student’s *t*-test (**A**–**C**) or two-way ANOVA with repeated measures (**D**,**E**).

Tomentosenol A (**1**) was previously reported to exhibit anti-bacterial activity against *S. aureus* with a minimum inhibitory concentration (MIC) of 4.74 μM and inhibited the growth of human cancer cell lines MCF-7, NCI-H460, SF-268, and HepG2 with IC_50_ values of 8.62–10.01 μM [21]. Torellianone A (**2**) has also been tested for anti-plasmodial activity against the 3D7 strain of *Plasmodium falciparum* parasite, but it did not show any inhibition up to a concentration of 40 μM [22]. This study focussed on the activity of tomentosenol A (**1**), which we found to inhibit the proliferation and migration of cultured primary human neonatal foreskin fibroblasts (NFF) and human immortalized keratinocytes (HaCaT). Compound **2** showed no significant effects on NFF cell viability, proliferation, or migration (Figures S22 and S23, Supporting Information). The effects of Compound **1** on cell viability were determined using an MTT assay and a sulforhodamine B (SRB) assay. Compound **1** had no significant effects on NFF or HaCaT cell viability in the concentration range of 0.75–6.25 µM with the MTT assay. However, MTT formazan production was reduced in NFFs by 30.0% and 43.5% and in HaCaTs by 28.6% and 52.6% after incubation with 12.5 µM and 25.0 µM Compound **1**, respectively (*p* < 0.05; Figure 4A,B). A similar result was obtained with the SRB assay, however a small, significant reduction in cell viability was also observed at the 6.25 µM concentration (Figure 4C). The effects of Compound **1** on NFF and HaCaT proliferation were examined over a 72-h incubation period, where it caused a dose-dependent inhibition of proliferation in NFF (Figure 4D) and HaCaT cells (Figure 4E).

**Figure 5:**
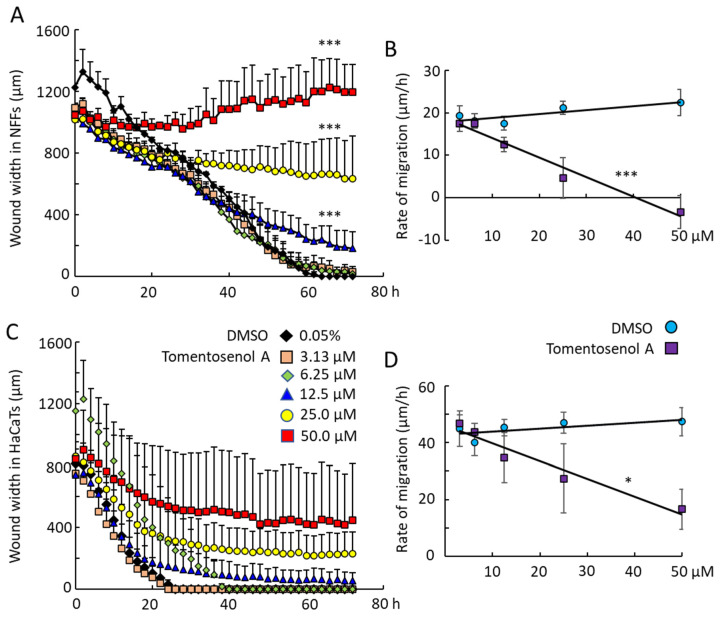
Effects of Compound **1** on scratch wound repopulation by neonatal human foreskin fibroblasts (NFFs) (**A**,**B**) and immortalized keratinocytes (HaCaTs) (**C**,**D**) incubated with Compound **1** for 72 h. Data were analysed for wound width (**A**,**C**) and rate of cell migration into the wound region (**B**,**D**). The small negative rate of migration observed for 50 μM Compound **1** with NFF indicates an expansion in wound area during the treatment period. Data shown are mean ± SEM for *n* = 3 independent experiments. * *p* < 0.05; *** *p* < 0.001, with comparison to DMSO control (**A**,**C**), 0.05% DMSO; (**B**,**D**), 0.0031−0.05% DMSO). Data were analysed using a two-way ANOVA with repeated measures.

Scratch wound assays were used to examine the effect of Compound **1** on NFF and HaCaT cell migration into a wound region. Compound **1** caused a concentration-dependent inhibition of wound repopulation by NFF (Figure 5A,B) and HaCaT cells (Figure 5C,D). Wound closure was achieved within 60 h for NFFs and within 26 h for HaCaTs exposed to the diluent (DMSO) control. The rates of wound closure were measured by examining the slope of the plot of wound width versus time and plotting this against the concentration of Compound **1**. The horizontal line for the DMSO control indicated that the vehicle had neither a stimulatory nor inhibitory effect on cell migration into the wound. In contrast, the line decreased with increasing concentrations of Compound **1**, indicating that the compound caused a concentration-dependent inhibition of cell migration. This inhibitory effect was significant for Compound **1** in NFFs (*p* < 0.001; Figure 5B) and HaCaTs (*p* < 0.05; Figure 5D). By limiting the migration of cells into the wound region, tomentosenol A (**1**) may suppress the fibrotic response.

**Figure 6:**
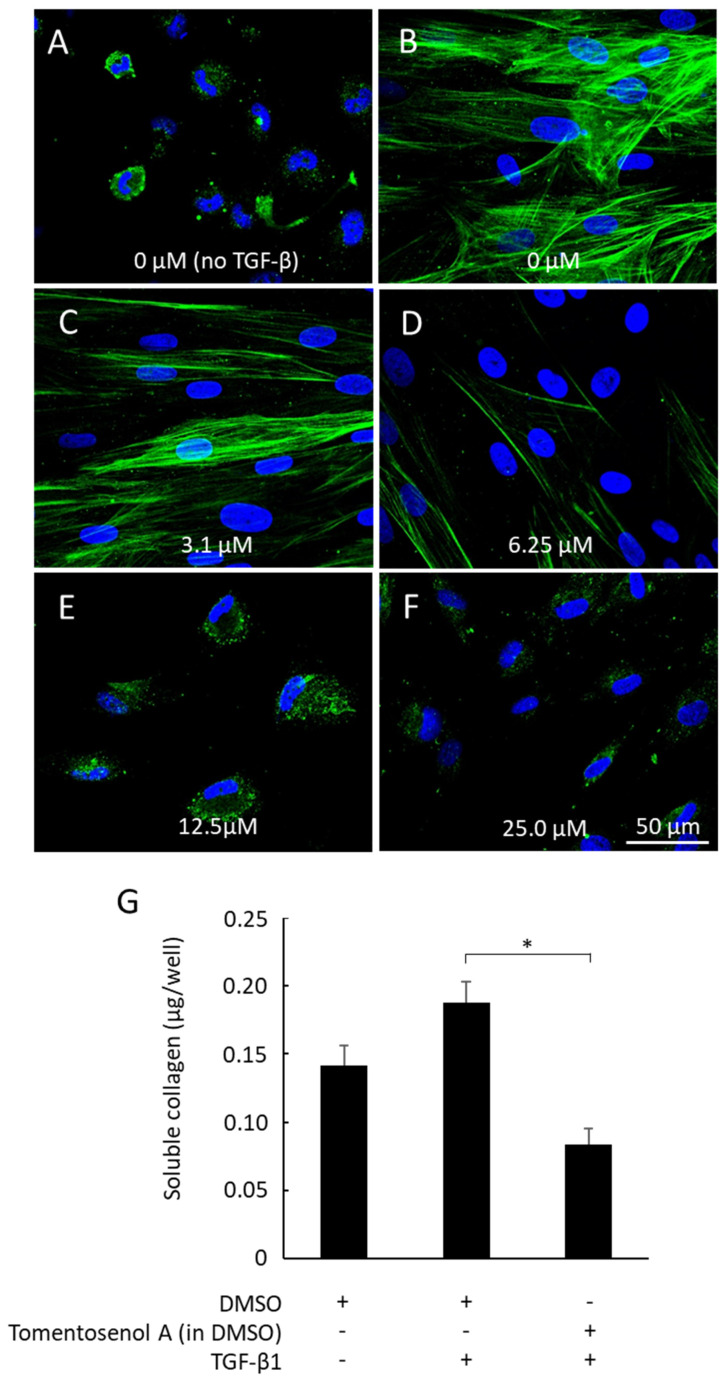
α−Smooth muscle actin (α−SMA) immunostaining and soluble collagen production in neonatal human foreskin fibroblasts (NFFs). Cells were incubated in the absence (**A**) or presence of 10 ng/mL TGF-β_1_ (**B**−**F**) and stained for α-SMA (green) and nuclei using DAPI (blue). Some cells were incubated without Compound **1** (**A**,**B**). Other cells were incubated with Compound **1** at concentrations of 3.1 µM (**C**), 6.25 µM (**D**), 12.5 µM (**E**), or 25 µM (**F**). Exposure of cells to TGF− β_1_-stimulated formation of α-SMA stress fibres, which was attenuated with 6.25 µM (**D**) or abolished with 12.5 µM (**E**) and 25 µM Compound **1** (**F**). Compound **1** (12.5 µM) also significantly inhibited TGF-β_1_-stimulated soluble collagen production (**G**). Data shown is mean ± SEM for *n* = 3 independent experiments. * *p* < 0.05, Data was analysed using a Student’s *t*-test.

During the wound healing process, fibroblasts are activated by TGF-β_1_ to differentiate into type I collagen-producing myofibroblasts [2,23], which are readily distinguished from non-differentiated cells by the expression of the cytoskeletal microfilament, α-smooth muscle actin (α-SMA). Agents that interfere with the TGF-β_1_ signalling pathway may provide anti-fibrotic benefit by inhibiting myofibroblast differentiation and collagen production [3]. This has been reported previously for small molecule meroterpenoids, petchiether A [24] and ganodercin M [25], isolated from the fruiting body of fungi, *Ganoderma petchii* and *G. cochlear*. The effect of Compound **1** on NFF-myofibroblast differentiation was examined by stimulating cells with TGF-β_1_ (10 ng/mL) and immunostaining for α-SMA. Fibroblasts remained non-differentiated when incubated without TGF-β_1_, as evidenced by the absence of α-SMA stress fibres (Figure 6A). In contrast, α-SMA fibres were detected in fibroblasts incubated with TGF-β_1_ (Figure 6B), consistent with myofibroblast differentiation. The α-SMA stress fibres remained visible in cells that were co-incubated with the lowest concentration of Compound **1** (3.1 µM) and TGF-β_1_ (Figure 6C). While staining for α-SMA was markedly diminished (6.25 µM; Figure 6D) or abolished (12.5–25 µM; Figure 6E,F) with higher concentrations of Compound **1**, cells remained viable throughout, thereby discounting any significant cytotoxic effects. In addition, TGF-β_1_-stimulated secretion of soluble collagen was supressed by co-incubation with Compound **1** (12.5 µM; *p* < 0.05, Figure 6G).

**Figure 7:**
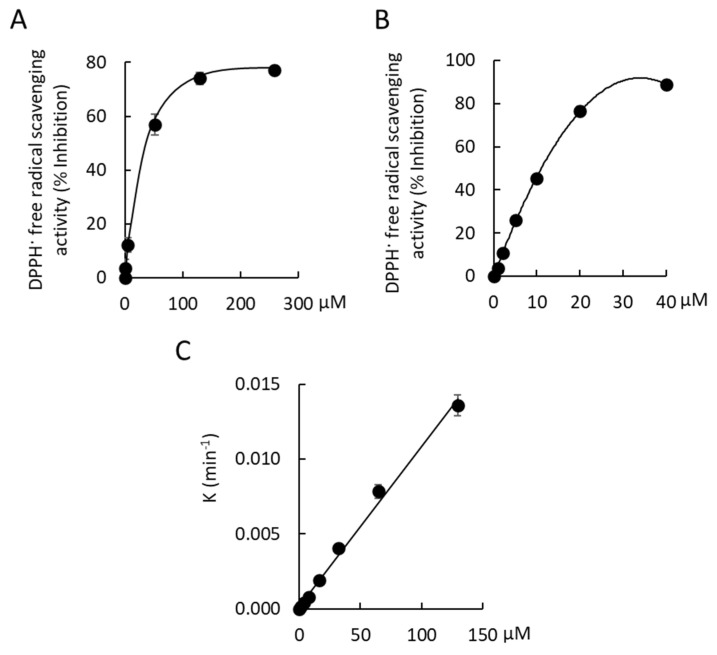
(**A**) 2,2-Diphenyl−1−picrylhydrazyl (DPPH·) free-radical scavenging by Compound **1** in a cell-free assay. Maximal free radical scavenging activity by Compound **1** was 77.1 ± 0.9% at 259 µM. The EC_50_ value for free radical scavenging activity was 44.7 ± 3.1 µM. (**B**) 2,2−Diphenyl−1−picrylhydrazyl (DPPH·) free-radical scavenging by gallic acid in a cell-free assay. Maximal free radical scavenging activity by gallic acid was 89.0 ± 0.2% at 40 µM. The EC_50_ value for free radical scavenging activity was 9.7 ± 0.5 µM. (**C**) The radical scavenging capacity of Compound **1** over 30 min at 21 °C was 1.0 × 10^−4^ μM^−1^.min^−1^ (R^2^ = 0.995). Data shown are mean ± SEM for *n* = 3 (**A**,**C**) and *n* = 6 (**B**) independent experiments.

Oxidative stress contributes to scar tissue formation and a higher concentration of reactive oxygen species (ROS) has been reported in keloid-derived fibroblasts, when compared with normal human dermal fibroblasts [26]. Dermal fibroblasts produce ROS via nicotinamide adenine dinucleotide phosphate (NADPH) oxidase, NOX2, in response to TGF-β_1_ [27]. The ROS were found to stimulate myofibroblast differentiation and increase collagen synthesis in keloid scar-derived, fibroblasts [27]. Taken together, these findings identify ROS as a potential target for the management of dermal fibrosis. We have previously ascribed anti-inflammatory and antioxidant activity to a crude methanolic extract of cerumen from *T. carbonaria* [15,18]. In the present study, we examined if tomentosenol A (**1**) contributes to this response using cell-free assays. Compound **1** (258 μM) had no effect on 5-lipoxygenase activity, an enzyme involved in the formation of the proinflammatory leukotriene, LTB_4_ (A_590_ nm; **1**, 0.70 ± 0.07; DMSO control, 0.71 ± 0.09). The free radical scavenging activity of 2,2-Diphenyl-1-picrylhydrazyl (DPPH·) was observed with **1**. Maximal free radical scavenging activity by Compound **1** was 77.1 ± 0.91% at a concentration of 259 µM (Figure 7A). The EC_50_ of Compound **1** was 44.7 ± 3.1 µM, which was ~8-fold less potent than a gallic acid positive control (EC_50_, 5.7 ± 0.4 µM). The radical scavenging capacity of Compound **1** was determined by measuring the slope of the plot of the first order rate constant, *K*, against the concentration of Compound **1** over a 30 min incubation at 21 °C (radical scavenging capacity, 1.0 × 10^−4^ μM^−1^ min^−1^ [R^2^ = 0.995; *n* = 3]; Figure 7B).

Our findings suggest that tomentosenol A (**1**) has potential anti-fibrotic activity in a cell-based assay system, at least in part, by inhibiting TGF-β_1_ signalling. TGF-β_1_ is known to induce NFF-myofibroblast differentiation via the activation of both Smad- and non–Smad dependent cell signalling pathways [28,29]. A recent study investigating the anti-fibrotic effects of the meroterpenoid, ganodercin M, found no effect on Smad2/3 phosphorylation in TGF-β_1_–stimulated rat renal proximal tubular cells (NRK-52E cells), suggesting a Smad-independent mechanism [25]. The precise mechanisms by which Compound **1** elicits its anti-fibrotic responses remain to be elucidated.

We used TGF-β_1_ to stimulate myofibroblast differentiation because it is the main activator of fibroblasts under pathophysiological conditions [3]. However, other profibrotic growth factors and cytokines, including platelet derived growth factor (PDGF), connective tissue growth factor (CTGF), fibroblast growth factor (FGF), and interleukins IL-4 and IL-13, are known to be expressed within the wound milieu. It is not yet known whether Compound **1** directly influences any of these cell signalling pathways, or if redundancy in fibrotic signalling pathways may limit the efficacy of Compound **1**. It is also important to note that wound healing and scar tissue formation are highly orchestrated events that require multiple intra- and inter-cellular interactions. It is therefore possible that the efficacy of Compound **1** might be different in a complex in vivo environment.

## 4. Conclusions

This study was successful in isolating a small molecule that has potential bioactivity in the prevention of scar tissue formation. The compound was identified as a meroterpenoid called tomentosenol A. Bioactivities associated with anti-scarring effects included the inhibition of fibroblast and keratinocyte migration, myofibroblast differentiation by TGF-β_1_ and TGF-β_1_-stimulated soluble collagen production and increased free radical scavenging activity. Activity of tomentosenol A at the higher concentrations (12.5 μM and 25.0 μM) was associated with a reduction in cell viability. Further in vitro mechanistic and in vivo studies using animal models and human subjects are required to investigate the full anti-fibrotic and anti-scarring potential of tomentosenol A.

## Data Availability

Data is contained within the article and supplementary material.

## Supplementary Materials

The following supporting information can be downloaded at: https://www.mdpi.com/article/10.3390/antiox11081604/s1, Table S1. NMR Data (^1^H 600 MHz, ^13^C 150 MHz) for **1**. Table S2. NMR Data (^1^H 600 MHz, ^13^C 150 MHz) for **2**. Figure S1. ^1^H Spectrum of **1** in CDCl_3_. Figure S2. ^13^C Spectrum of **1** in CDCl_3_. Figure S3. HSQC Spectrum of **1** in CDCl_3_. Figure S4. COSY Spectrum of **1** in CDCl_3_. Figure S5. HMBC Spectrum of **1** in CDCl_3_. Figure S6. ^1^H Spectrum of **1** in MeOH-*d_4_*. Figure S7. ^13^C Spectrum of **1** in MeOH-*d_4_*. Figure S8. HSQC Spectrum of **1** in MeOH-*d_4_*. Figure S9. COSY Spectrum of **1** in MeOH-*d_4_*. Figure S10. HSQC_TOCSY Spectrum of **1** in MeOH-*d_4_;* Figure S11. HMBC Spectrum of **1** in MeOH-*d_4_*. Figure S12. ^1^H Spectrum of **2** in DMSO-*d_6_*; Figure S13. ^13^C Spectrum of **2** in DMSO-*d_6_*. Figure S14. HSQC Spectrum of **2** in DMSO-*d_6_*. Figure S15. COSY Spectrum of **2** in DMSO-*d_6_*. Figure S16. HMBC Spectrum of **2** in DMSO-*d_6_*. Figure S17. 1D ROESY Spectrum of **2** in DMSO-*d_6_* (irradiated at 5.70 ppm). Figure S18. 1D ROESY Spectrum of **2** in DMSO-*d_6_* (irradiated at 5.10 ppm). Figure S19. 1D ROESY Spectrum of **2** in DMSO-*d_6_* (irradiated at 2.17 ppm). Figure S20. ^1^H Spectrum of **2** in CDCl_3_. Figure S21. Cell viability assessed using the MTT assay after 24 h incubation of NFFs with 0.75–25 μM of **2** (A, *n* = 8). NFF proliferation during 72 h treatment with **2** (B; **2**, *n* = 2; DMSO, *n* = 3). Data are mean ± SEM. Figure S22. Effects of **2** (*n* = 2) or DMSO (*n* = 3) on wound repopulation by NFFs incubated for 72 h (*n* = 2). Data was analysed for wound width (A) and rate of cell migration into the wound region (B).
