# Supplementary material for: Anti-Fibrotic Potential of Tomentosenol A, a Constituent of Cerumen from the Australian Native Stingless Bee, Tetragonula carbonaria
Source: Antioxidants (Basel). 2022 Aug 19;11(8):1604. doi: 10.3390/antiox11081604 (PMC9404848; doi:10.3390/antiox11081604)

Anti-fibrotic Potential of Tomentosenol A, a Constituent of Cerumen from the Australian Stingless Bee *Tetragonula carbonaria*

Supporting Information

Table S1. NMR Data (^{1}H 600 MHz, ^{13}C 150 MHz) for **1**

Table S2. NMR Data (^{1}H 600 MHz, ^{13}C 150 MHz) for **2**

Figure S1. ^{1}H Spectrum of **1** in $CDCl_3$

Figure S2. ^{13}C Spectrum of **1** in $CDCl_3$

Figure S3. HSQC Spectrum of **1** in $CDCl_3$

Figure S4. COSY Spectrum of **1** in $CDCl_3$

Figure S5. HMBC Spectrum of **1** in $CDCl_3$

Figure S6. ^{1}H Spectrum of **1** in MeOH-d_4

Figure S7. ^{13}C Spectrum of **1** in MeOH-d_4

Figure S8. HSQC Spectrum of **1** in MeOH-d_4

Figure S9. COSY Spectrum of **1** in MeOH-d_4

Figure S10. HSQC_TOCSY Spectrum of **1** in MeOH-d_4

Figure S11. HMBC Spectrum of **1** in MeOH-d_4

Figure S12. ^{1}H Spectrum of **2** in DMSO-d_6

Figure S13. ^{13}C Spectrum of **2** in DMSO-d_6

Figure S14. HSQC Spectrum of **2** in DMSO-d_6

Figure S15. COSY Spectrum of **2** in DMSO-d_6

Figure S16. HMBC Spectrum of **2** in DMSO-d_6

Figure S17. 1D ROESY Spectrum of **2** in DMSO-d_6 (irradiated at 5.70 ppm)

Figure S18. 1D ROESY Spectrum of **2** in DMSO-d_6 (irradiated at 5.10 ppm)

Figure S19. 1D ROESY Spectrum of **2** in DMSO-d_6 (irradiated at 2.17 ppm)

Figure S20. ^{1}H Spectrum of **2** in $CDCl_3$

Figure S21. Cell viability assessed using the MTT assay after 24h incubation of NFFs with 0.75-25 μM of **2** (A, n=8). NFF proliferation during 72 h treatment with **2** (B; **2**, n=2; DMSO, n=3). Data are mean±SEM.

Figure S22. Effects of **2** (n=2) or DMSO (n=3) on wound repopulation by NFFs incubated for 72 h (n=2). Data was analysed for wound width (A) and rate of cell migration into the wound region (B).

Table S1. NMR Data (^{1}H 600 MHz, ^{13}C 150 MHz) for **1**

	**Tomentosenol A** [a,b]		**1A** [b]		**1B** [b]		**1A** [c]	
Pos.	δ_C	δ_H	δ_C	δ_H	δ_C	δ_H	δ_C	δ_H
2(2')	146.3, C		146.4, C		147.5, C		149.3, C	
3	41.2, CH_2	2.49, m	41.4, CH_2	2.47, m	39.1, CH_2	2.33 [e]	42.2, CH_2	2.59, dd (10.8, 13.2)
		2.06, m		2.09, m		2.12 [e]		1.96, m
4	31.1, CH	3.02, brs	31.4, CH	3.00, brs	31.3, CH	2.48 [e]	31.9, CH	3.28, m
4a	114.3, C		114.4, C		61.1, CH	3.74, d (2.4)	115.9, CH	
5	198.0, C		198.0, C		205.4, C		[d]	
6	55.7, C		55.9, C		59.0, C		53.2, C	
7	212.9, C		213.0, C		210.7, C		215.1, C	
8	47.7, C		47.9, C		59.1, C		51.4, C	
8a	170.1, C		170.2, C		205.7, C		[d]	
9	25.2, CH_3	1.46, s	25.1, CH_3	1.42, s	22.6, CH_3	1.33, s	25.8, CH_3	1.33, s
10	24.9, CH_3	1.43, s	26.5, CH_3	1.41, s	23.2, CH_3	1.29, s	24.6, CH_3	1.32, s
11	25.6, CH_3	1.33, s	23.7, CH_3	1.30, s	21.8, CH_3	1.33, s	25.8, CH_3	1.33, s
12	23.6, CH_3	1.32, s	25.7, CH_3	1.32, s	23.5, CH_3	1.27, s	24.6, CH_3	1.32, s
1'	45.3, CH	2.08, m	45.4, CH	2.08, m	45.2, CH	2.05, m	42.1, CH	2.00, m
3'	118.3, CH	5.12, s	118.4, CH	5.10, s	119.2, CH	5.16, s	117.9, CH	5.03, s
4'	31.3, CH_2	2.13, m	31.5, CH_2	2.17, m	29.8, CH_2	1.25, m	32.3, CH_2	2.13, t (2.4)
5'	40.7, CH	2.04, m	40.9, CH	2.03, m	40.9, CH	2.03, m	42.1, CH	2.00, m
6'	38.2, C		38.3, C		38.3, C		38.9, C	
7'	31.8, CH_2	2.33, dt (5.6, 8.5)	31.9, CH_2	2.33 [e]	32.2, CH_2	2.35 [e]	32.9, CH_2	2.31, dt (5.4, 8.4)
		0.93, d (8.5)		0.92 [e]		0.85 [e]		0.97, d (8.4)
8'	21.0, CH_3	1.26, s	21.2, CH_3	0.81, s	21.2, CH_3	0.82, s	21.5, CH_3	0.83, s
9'	26.3, CH_3	0.83, s	26.5, CH_3	1.25, s	26.4, CH3	1.26, s	26.9, CH_3	1.27, s
1''	42.9, CH_2	1.69, m	43.1, CH_2	1.68, m	40.0, CH_2	1.61 [e]	43.9, CH_2	1.82, m
		1.41 [e]		1.41 [e]		1.00, m		1.23, m
2''	26.5, CH	1.41 [e]	26.7, CH	1.42 [e]	25.9, CH	1.63, m	27.8, CH	1.38, m
3''	23.4, CH_3	0.89, d (6.0)	23.6, CH_3	0.87, d (5.4)	21.6, CH_3	0.93, d (6.6)	24.2, CH_3	0.84, d (6.6)
4''	22.3, CH_3	0.87, d (6.0)	22.4, CH_3	0.85, d (5.4)	23.9, CH_3	0.88, d (6.0)	22.9, CH_3	0.86, d (6.6)
8a-OH		5.72, s		5.65, brs				

[a] Referenced Liu, H.-X.; Zhang, W.-M.; Xu, Z.-F.; Chen, Y.-C.; Tan, H.-B.; Qiu, S.-X., *RSC Adv.* **2016,** 6, 25882-25886

[b] Recorded in $CDCl_3$

[c] Recorded in CD_3OD

[d] Not observed

[e] Overlapped signals

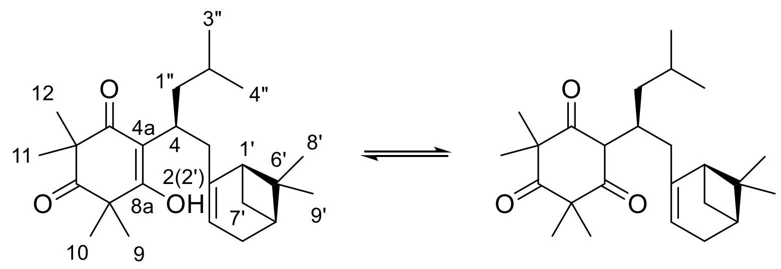

1A **1B**

Table S2. NMR Data (^{1}H 600 MHz, ^{13}C 150 MHz) for **2**

Pos.	Torellianone A [a,b]	Torellianone B [a,b]	**2** [b]	**2** [c]			
	δ_H	δ_H	δ_H	δ_H	δ_C	ROESY	HMBC
1a					165.2, C		
2					46.8, C		
3					210.7, C		
4					55.7, C		
5					196.1, C		
5a					111.9, C		
6	5.283, s	5.275, s	5.280, s	5.10, s	32.3, CH	16-OH, 2'/6'	5, 1a, 8, 16, 1', 2', 6', 5a, 7
7					105.3, C		
8					154.0, C		
9					103.6, C		
10					157.9, C		
12	5.411, dd (3.1, 12.7)	5.406, dd (13.2, 2.9)	5.411, dd (3.0, 12.6)	5.70, d (12.0)	78.3, CH	13b	14, 2", 6", 1", 13
13	α: 3.05, dd (12.7, 17.2)	3.02, dd (13.2, 17.2)	3.05, dd (13.2, 16.8)	3.30, dd (12.0, 16.8)	41.9, CH2	2"/6"	14, 12, 1"
	β: 2.857, dd (3.1, 17.2)	2.847, dd (17.2, 2.9)	2.856, dd (2.4, 17.4)	2.93, d (17.4)			14, 15, 1"
14					198.0, C		
15					104.9, C		
16					157.2, C		
17	2.22, s	2.22, s	2.22, s	2.17, s	7.5, CH3	12, 2"/6"	8, 9, 10
18	1.13, s	1.13, s	1.13, s	1.04, s	24.3, CH3		3, 4, 5, 19
19	1.34, s	1.34, s	1.34, s	1.24, s	22.8, CH3		3, 4, 5, 18
20	1.546, s	1.55, s	1.547, s	1.49, s	24.4, CH3		1a, 2, 3, 21
21	1.65, s	1.65, s	1.65, s	1.60, s	24.9, CH3		1a, 2, 3, 20
1'					143.7, C		
2'/6'	7.31, dd (1.6, 7.3)	7.30, dd (J = 1.6, 7.3)	7.31, d (7.2)	7.23, m	127.7, CH x 2	6	6, 4'
3'/5'	7.24, t (7.3)	7.22, t (J = 7.3)	7.24, t (7.8)	7.25, m	128.2, CH x 2		1'
4'	7.15, tt (1.2, 7.3)	7.14, tt (J = 1.2, 7.3)	7.15, t (7.2)	7.14, t (7.2)	126.4, CH		1', 2', 6'
1"					138.5, C		
2"/6"	7.45, m	7.45, m	7.45, m	7.52, d (7.2)	126.4, CH x 2		12, 4"
3"/5"	7.45, m	7.45, m	7.45, m	7.45, t (7.2)	128.7, CH x 2		1", 2", 6"
4"	7.41, m	7.41, m	7.41, m	7.40, t (7.2)	128.6, CH		2", 6"
16-OH	12.08, s	12.07, s	12.09, s	12.30, s		6	7, 15, 16, 14_w

[a] Referenced Senadeera, S. P.; Lucantoni, L.; Duffy, S.; Avery, V. M.; Carroll, A. R., *J. Nat. Prod.* **2018,** 81, 1588-1597

[b] Recorded in $CDCl_3$

[c] Recorded in DMSO-d_6

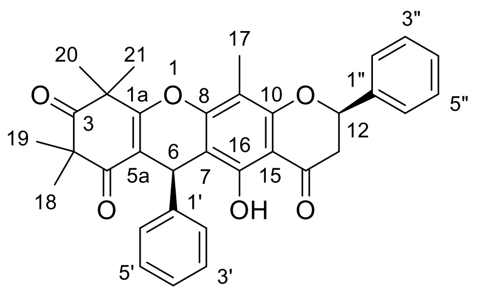

2

Figure S1. ^{1}H Spectrum of **1** in $CDCl_3$

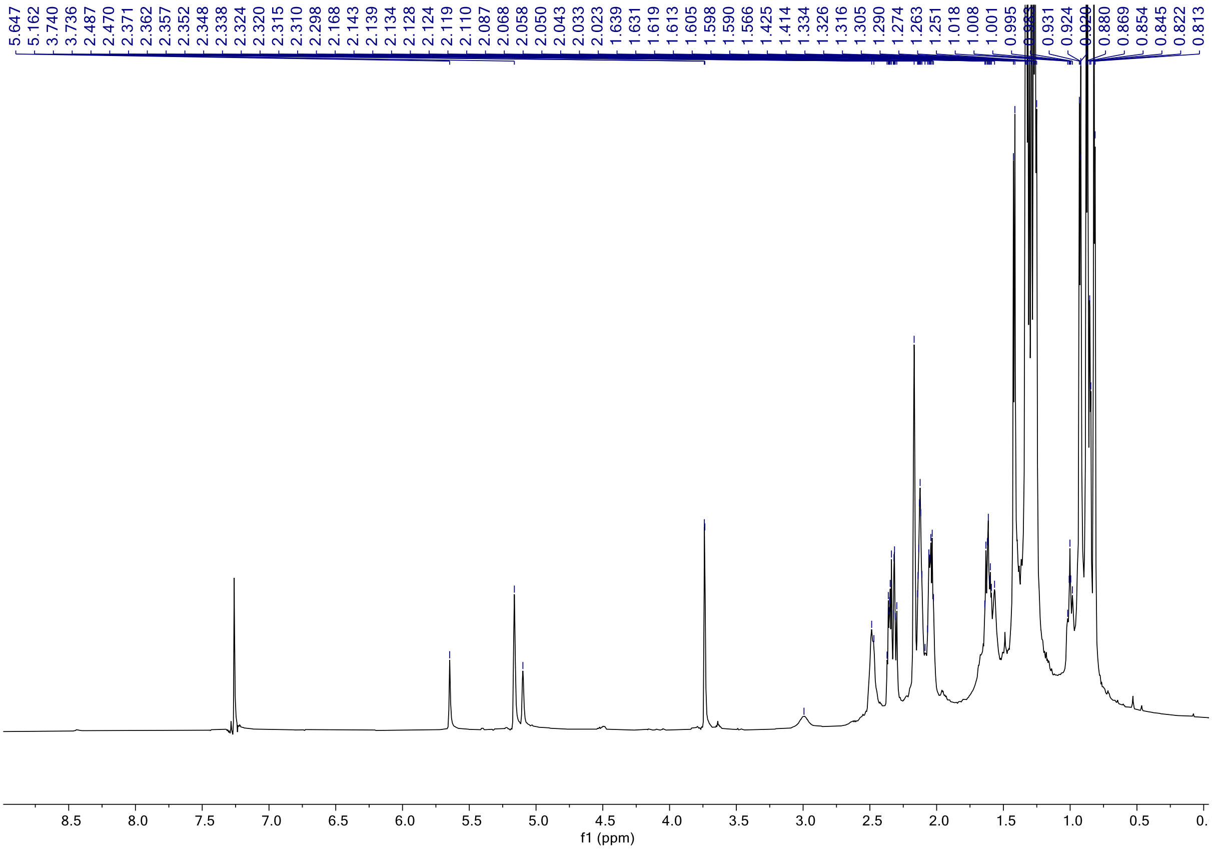

Figure S2. ^{13}C Spectrum of **1** in $CDCl_3$

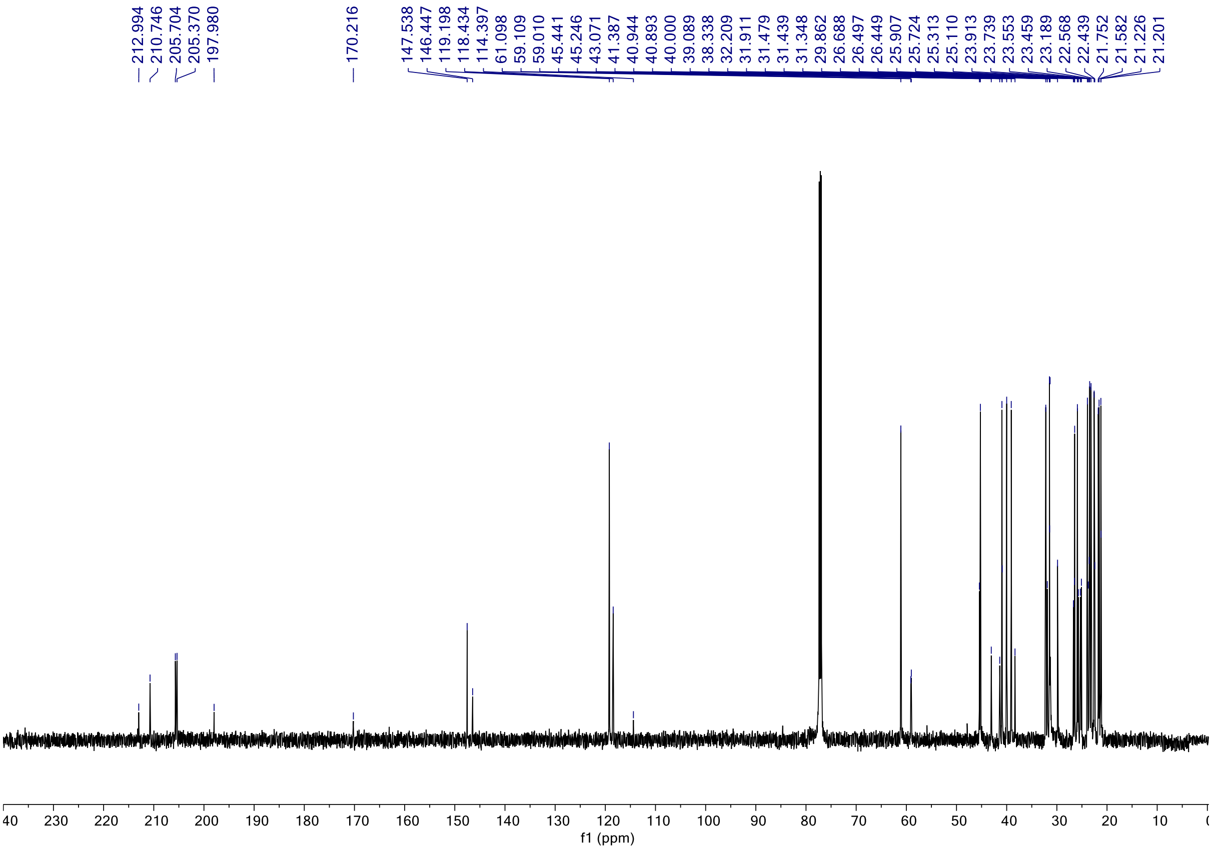

Figure S3. HSQC Spectrum of **1** in $CDCl_3$

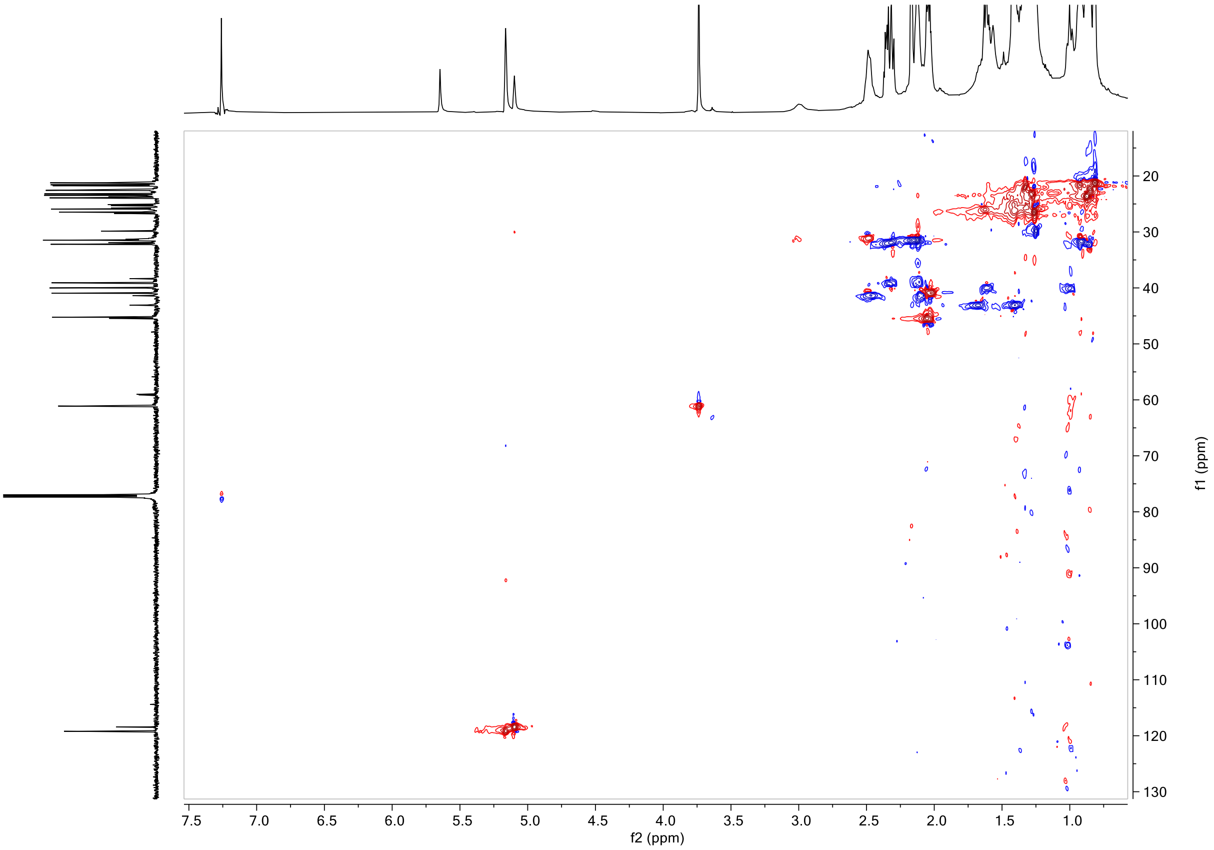

Figure S4. COSY Spectrum of **1** in $CDCl_3$

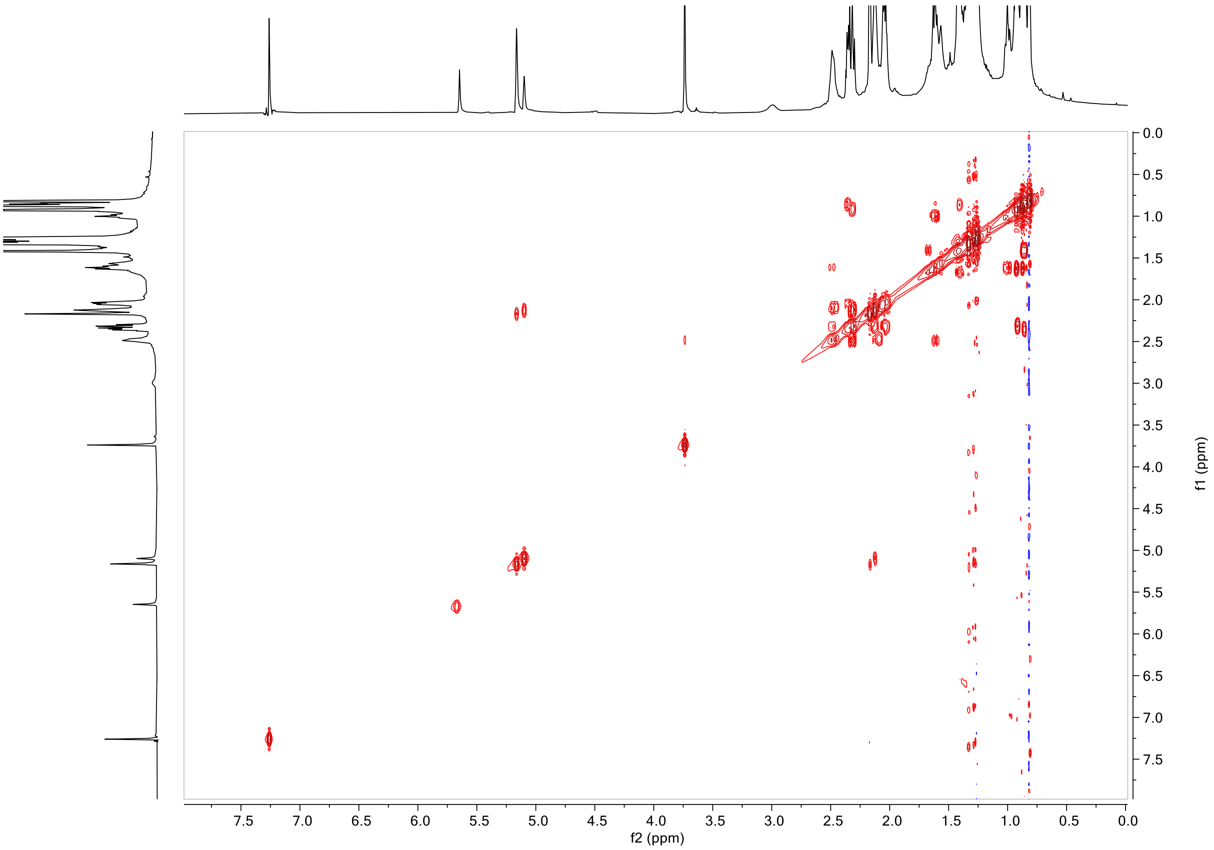

Figure S5. HMBC Spectrum of **1** in $CDCl_3$

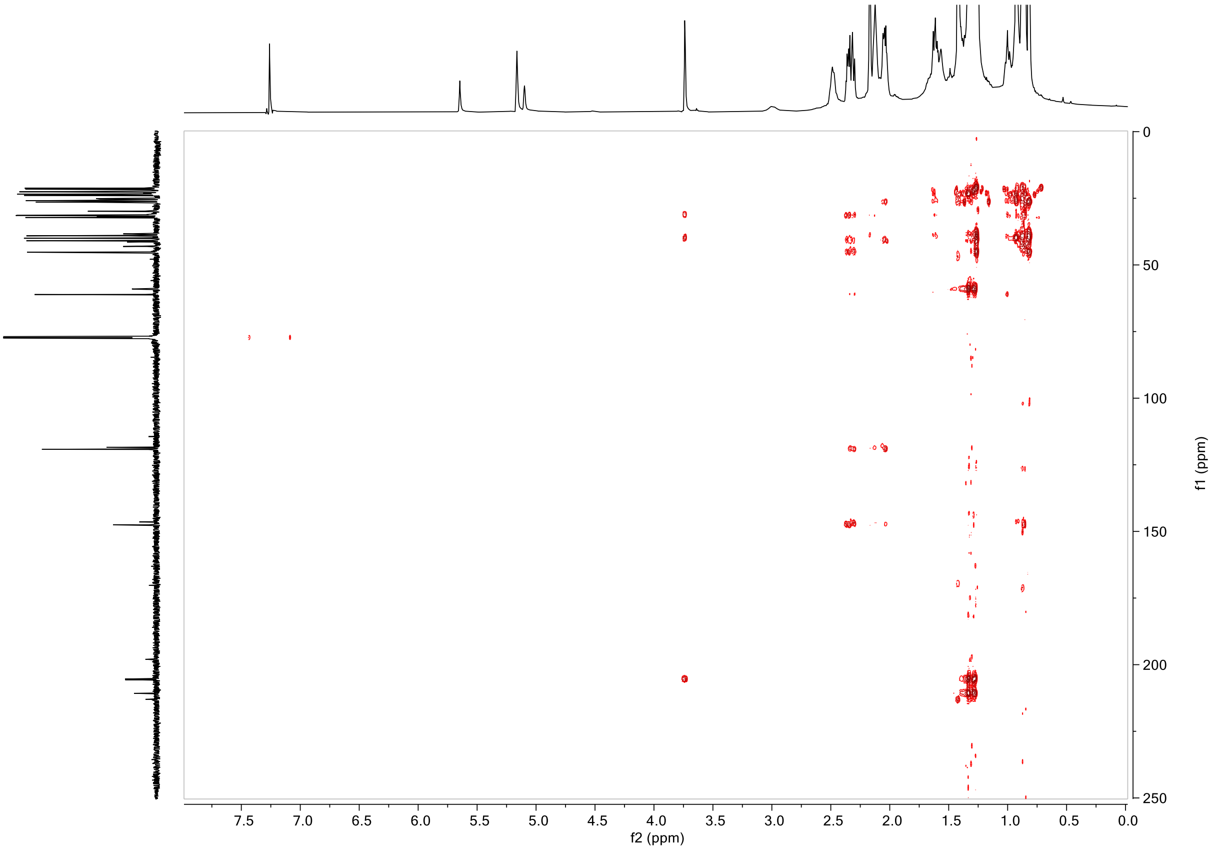

Figure S6. ^{1}H Spectrum of **1** in MeOH-d_4

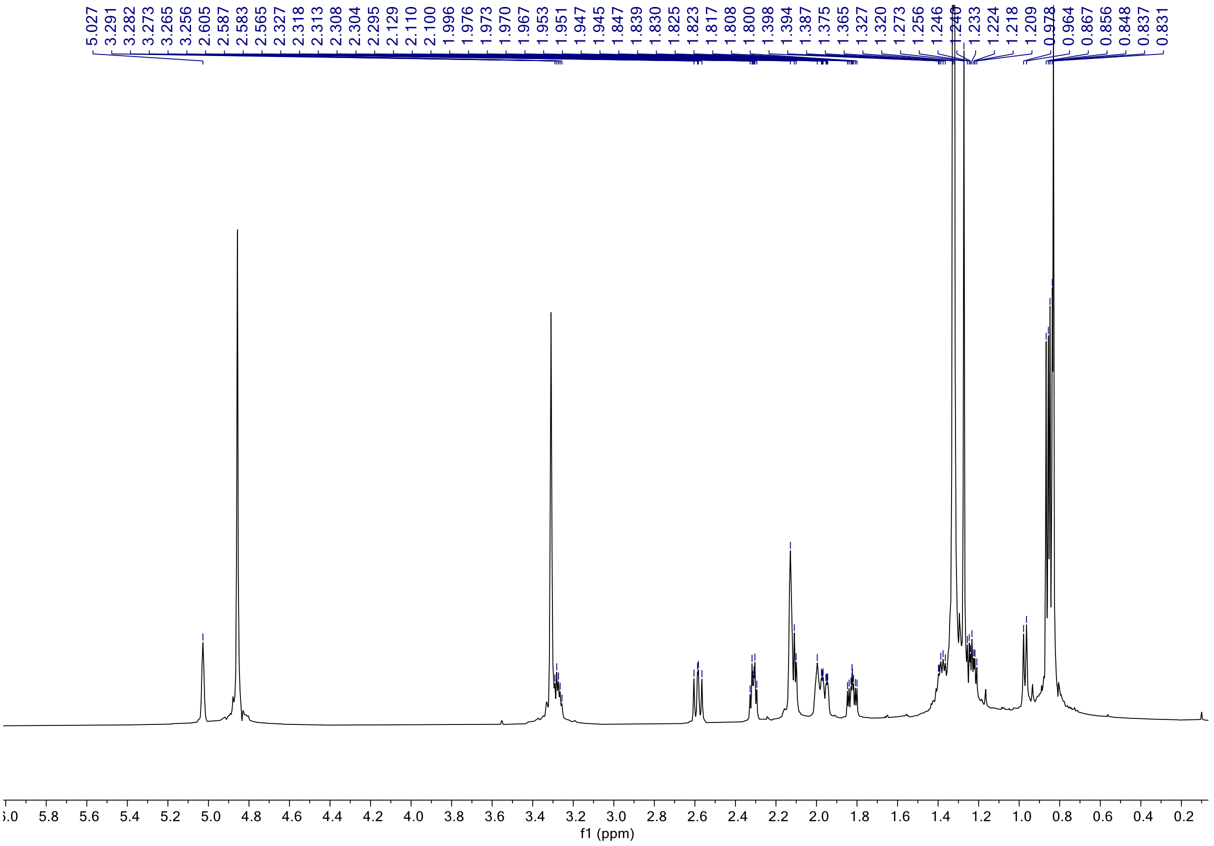

Figure S7. ^{13}C Spectrum of **1** in MeOH-d_4

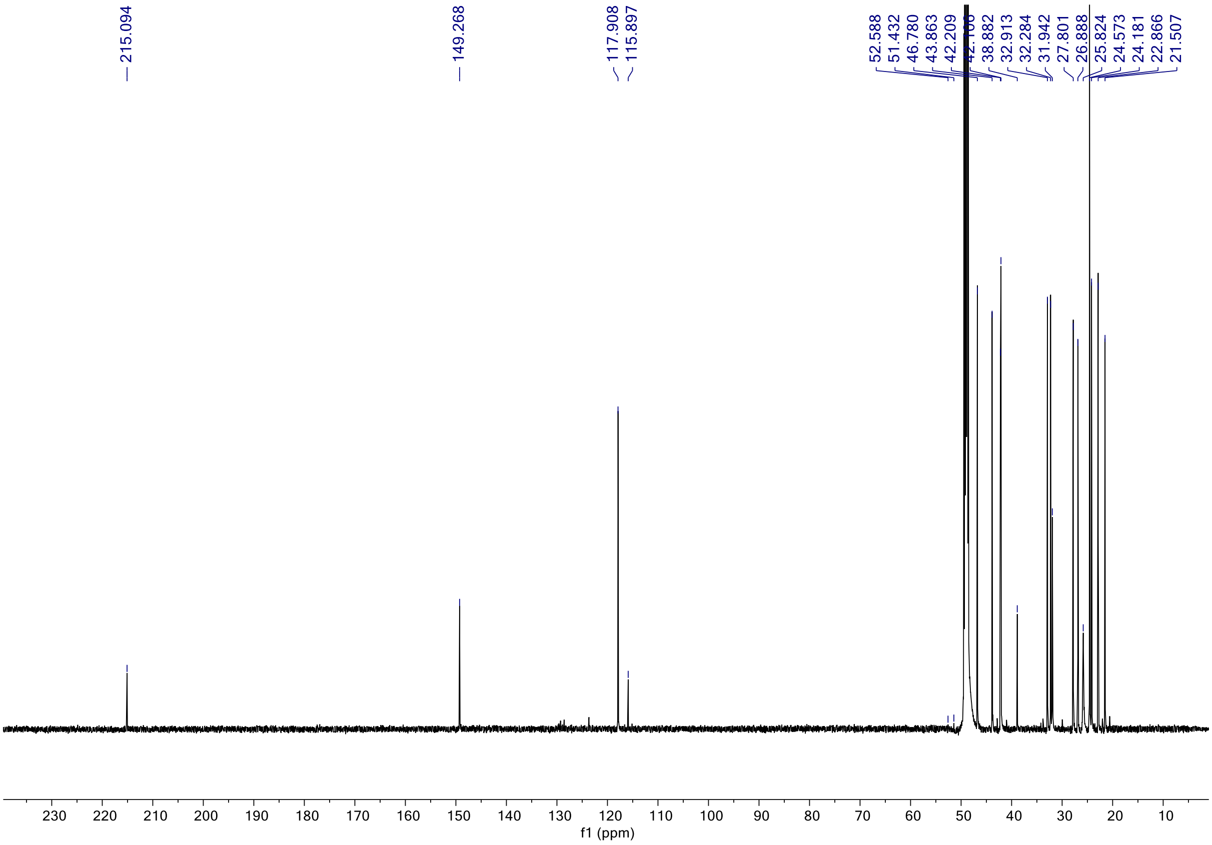

Figure S8. HSQC Spectrum of **1** in MeOH-d_4

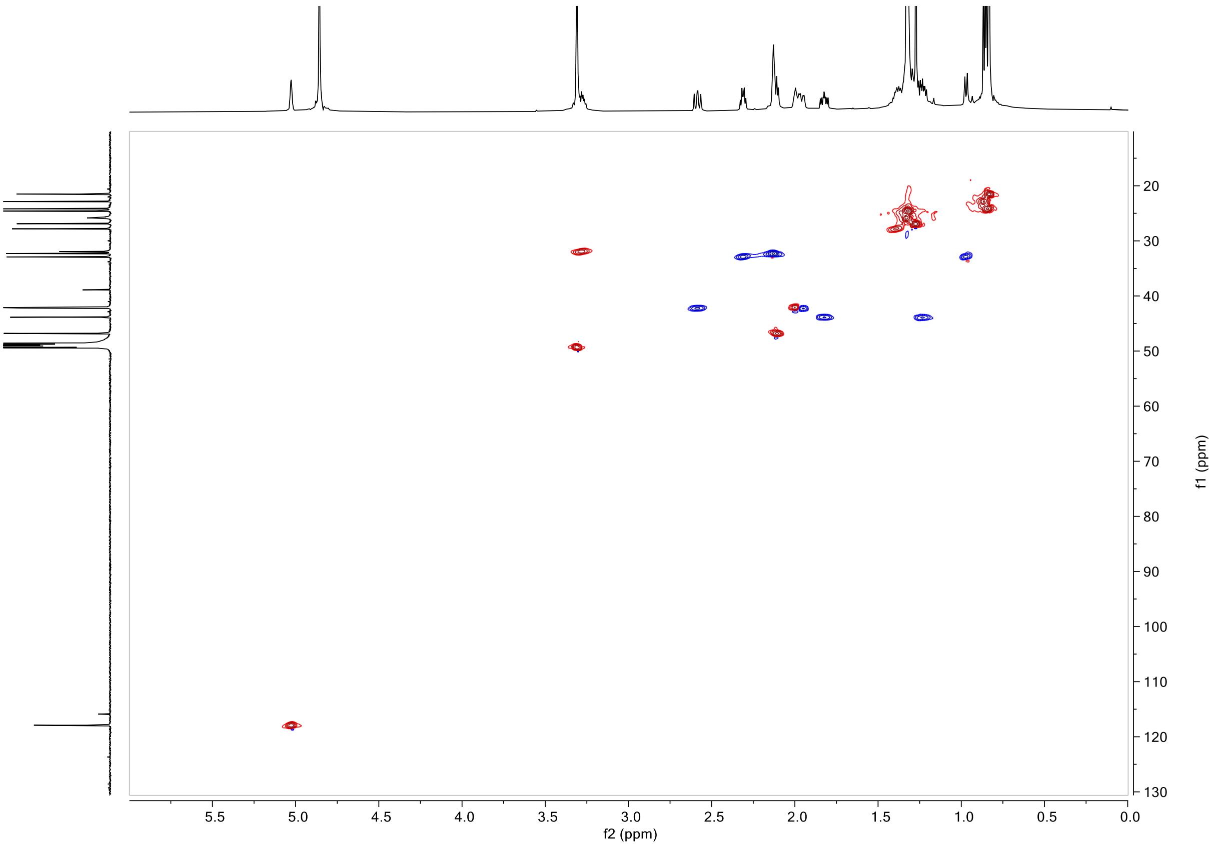

Figure S9. COSY Spectrum of **1** in MeOH-d_4

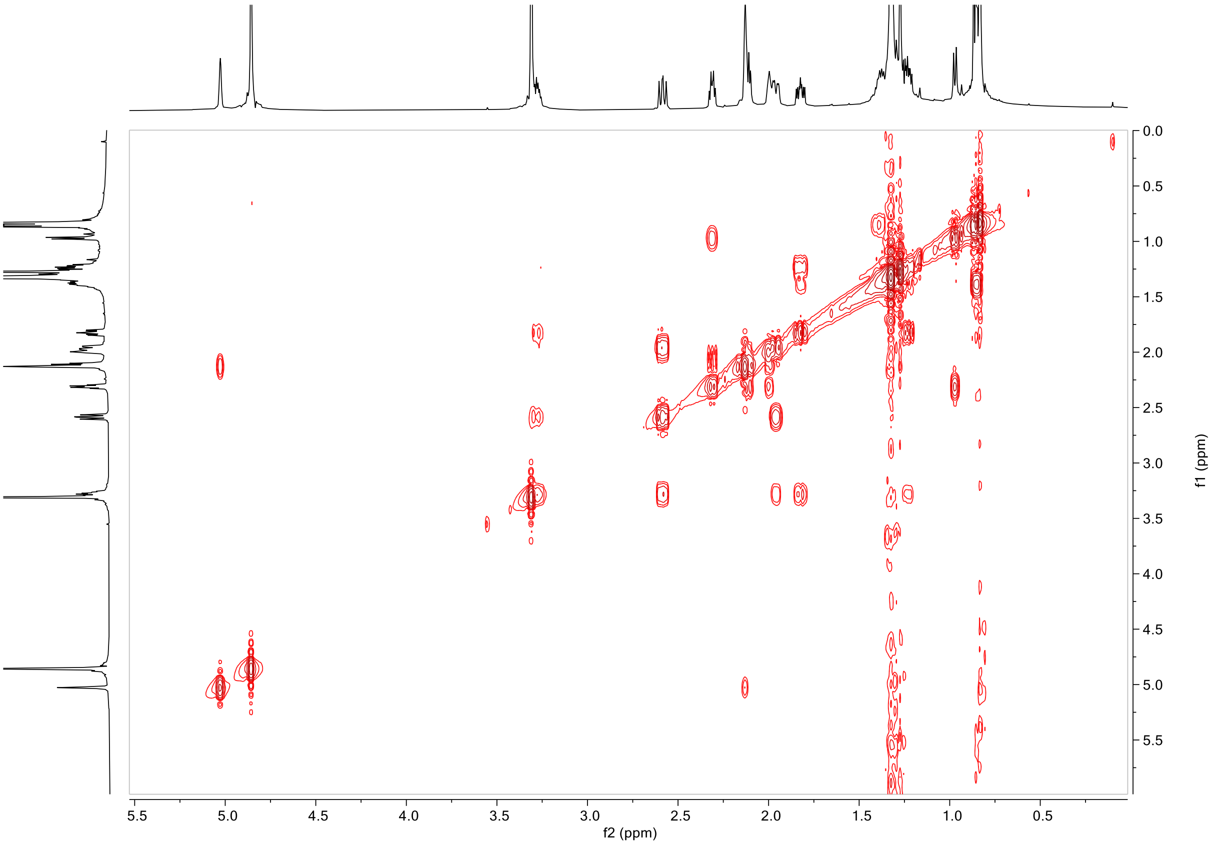

Figure S10. HSQC_TOCSY Spectrum of **1** in MeOH-d_4

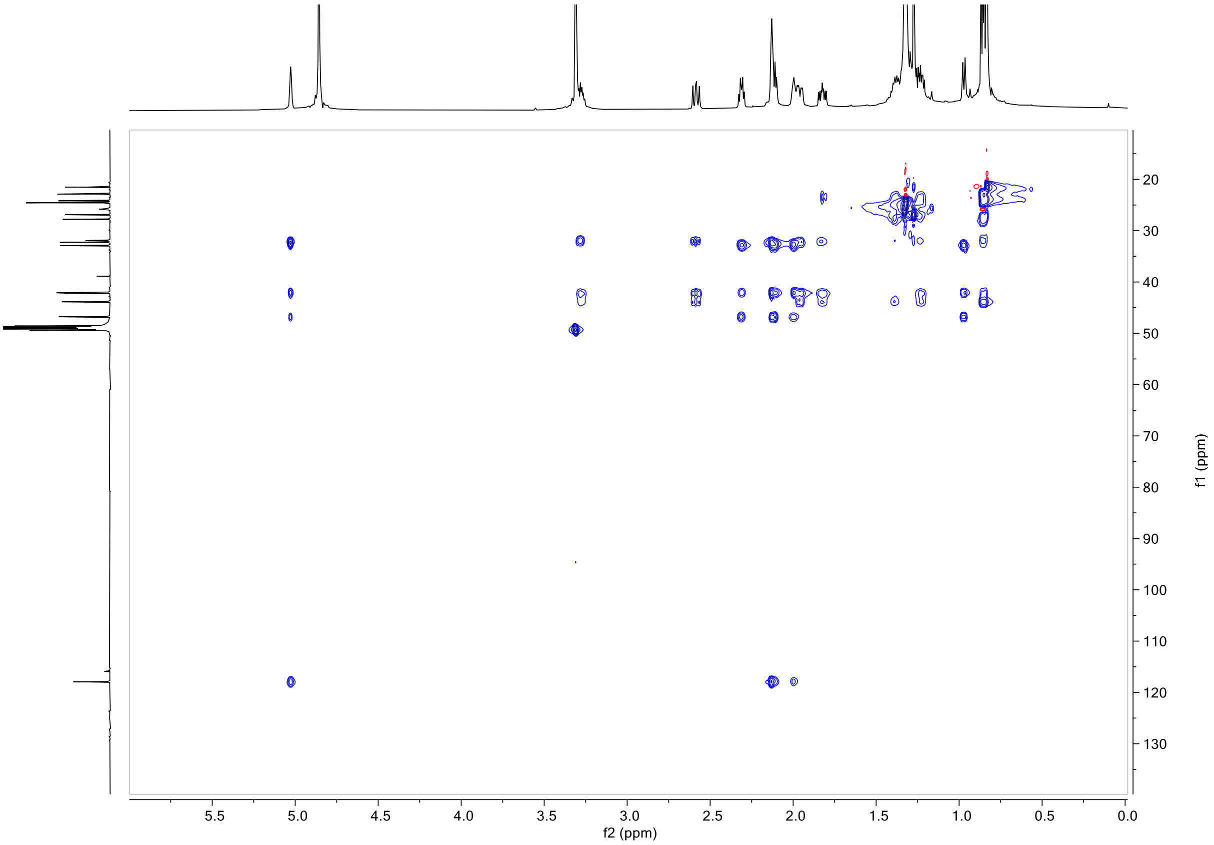

Figure S11. HMBC Spectrum of **1** in MeOH-d_4

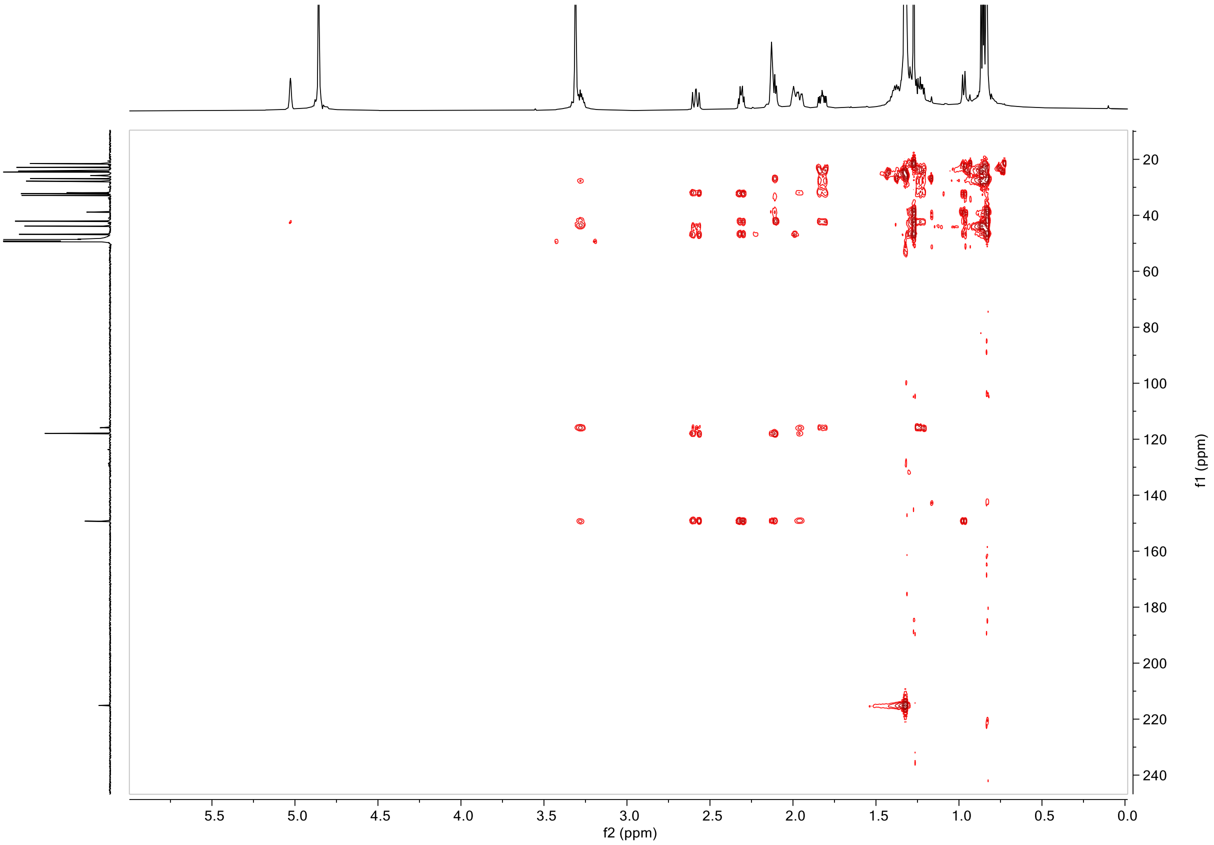

Figure S12. ^{1}H Spectrum of **2** in DMSO-d_6

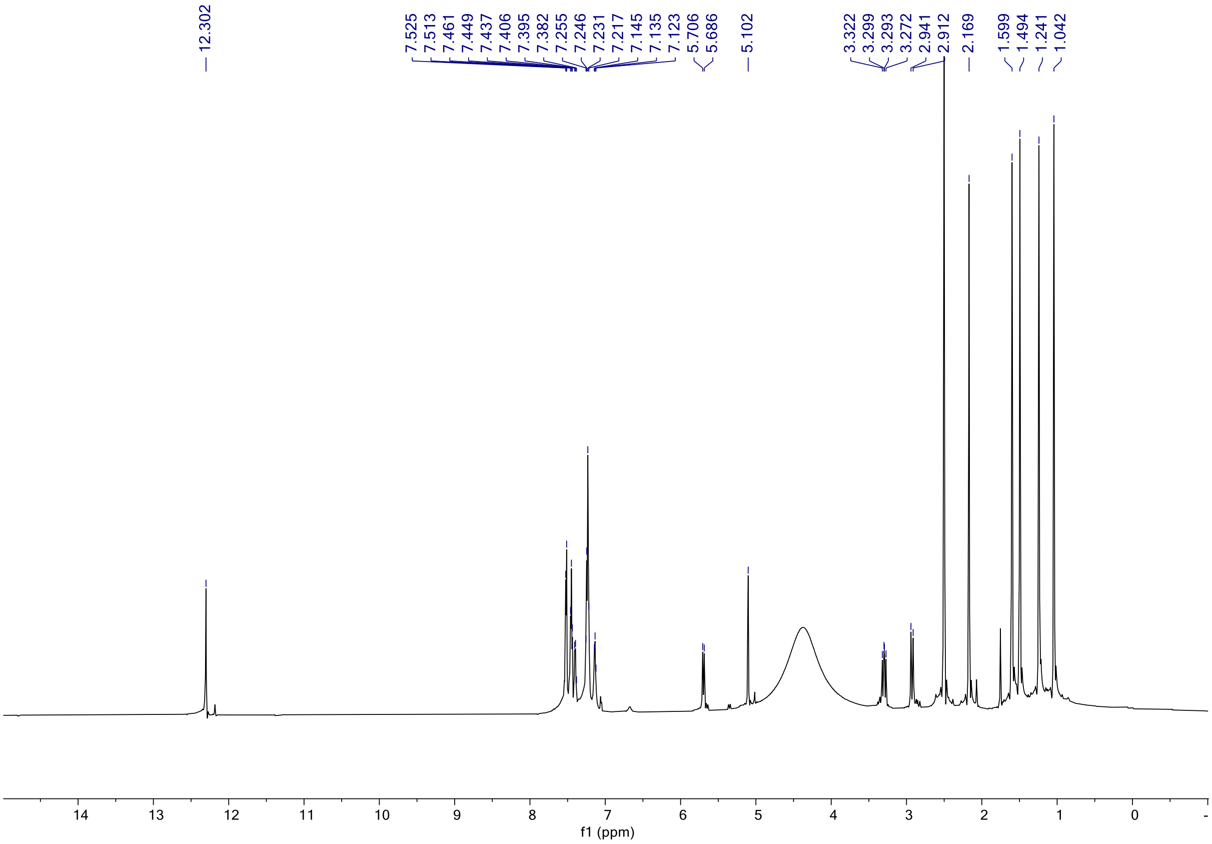

Figure S13. ^{13}C Spectrum of **2** in DMSO-d_6

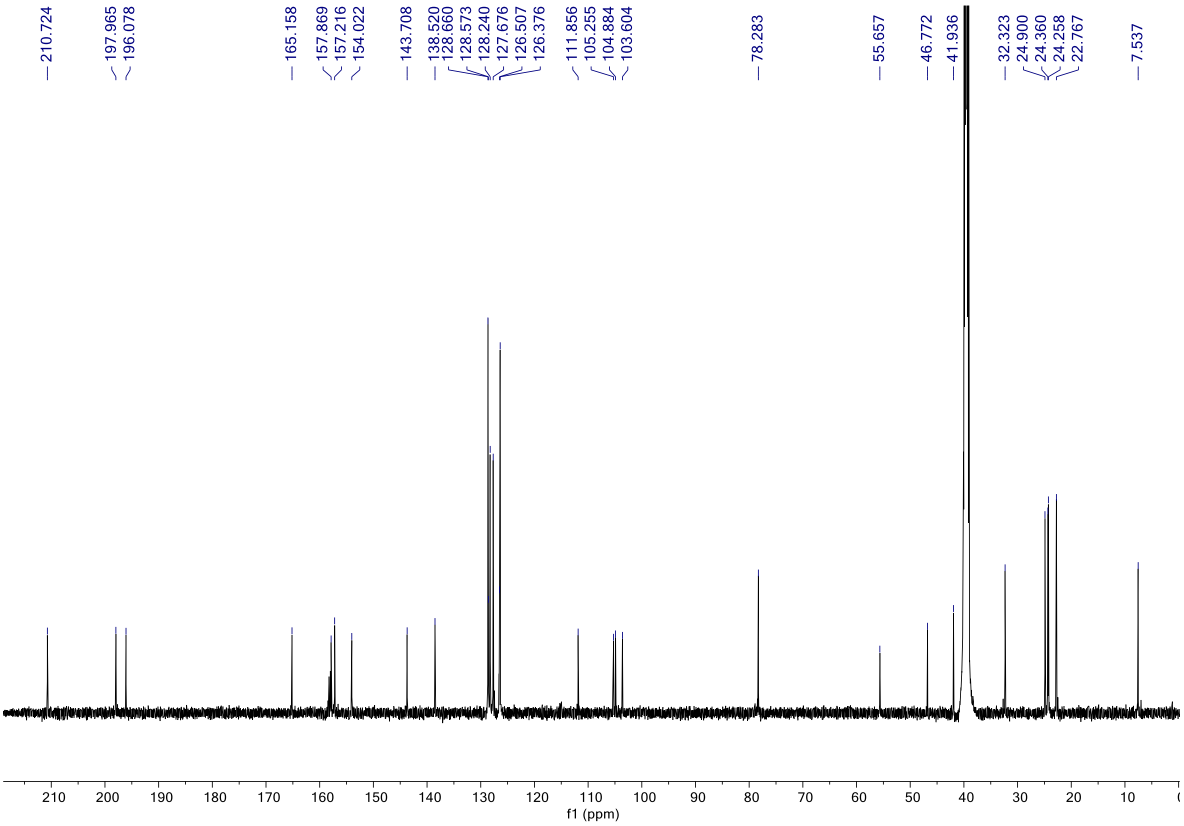

Figure S14. HSQC Spectrum of **2** in DMSO-d_6

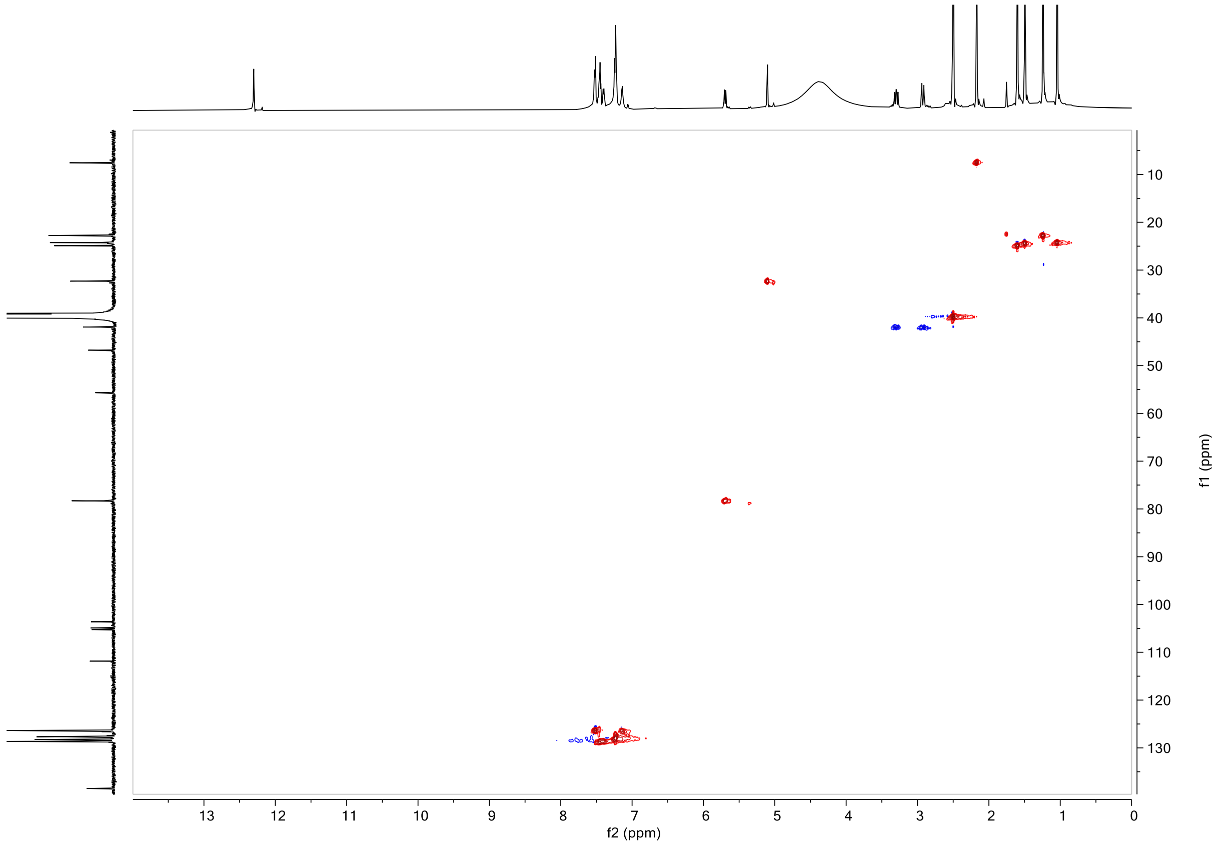

Figure S15. COSY Spectrum of **2** in DMSO-d_6

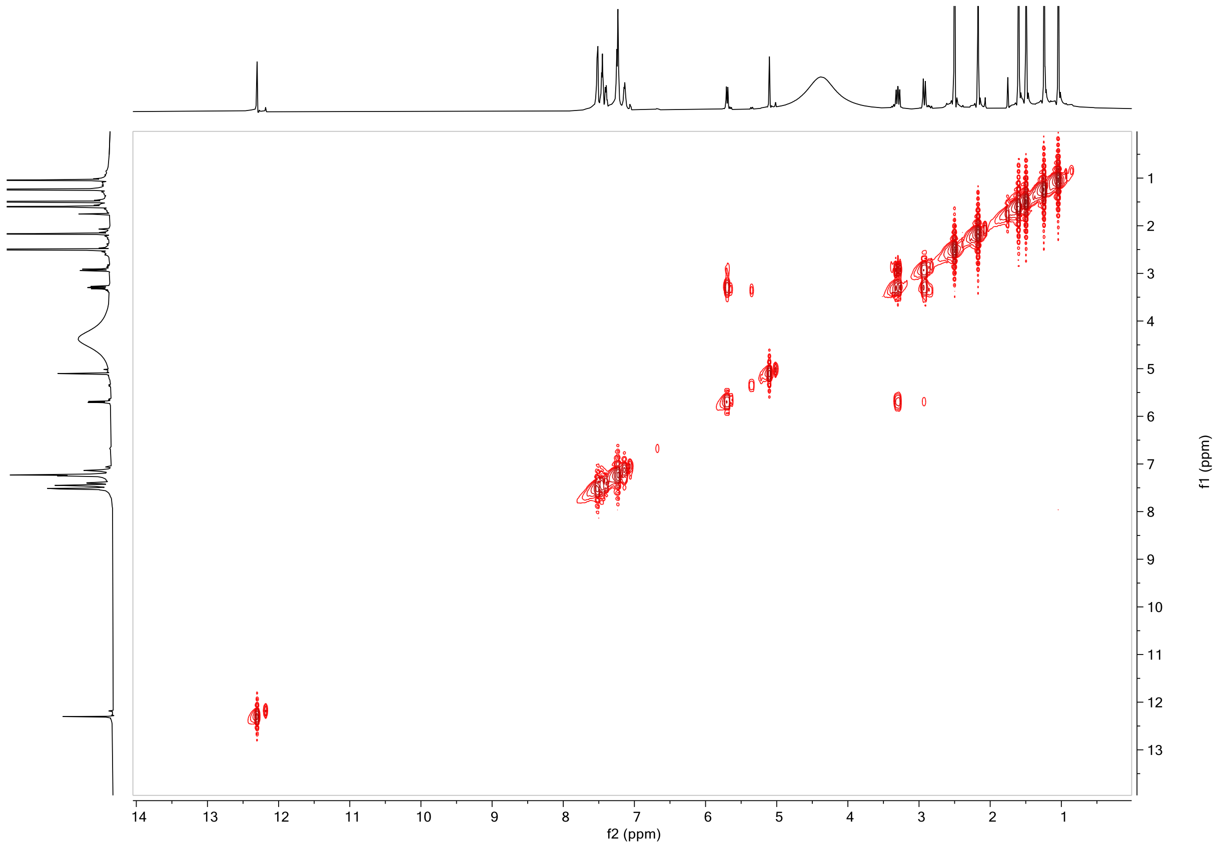

Figure S16. HMBC Spectrum of **2** in DMSO-d_6

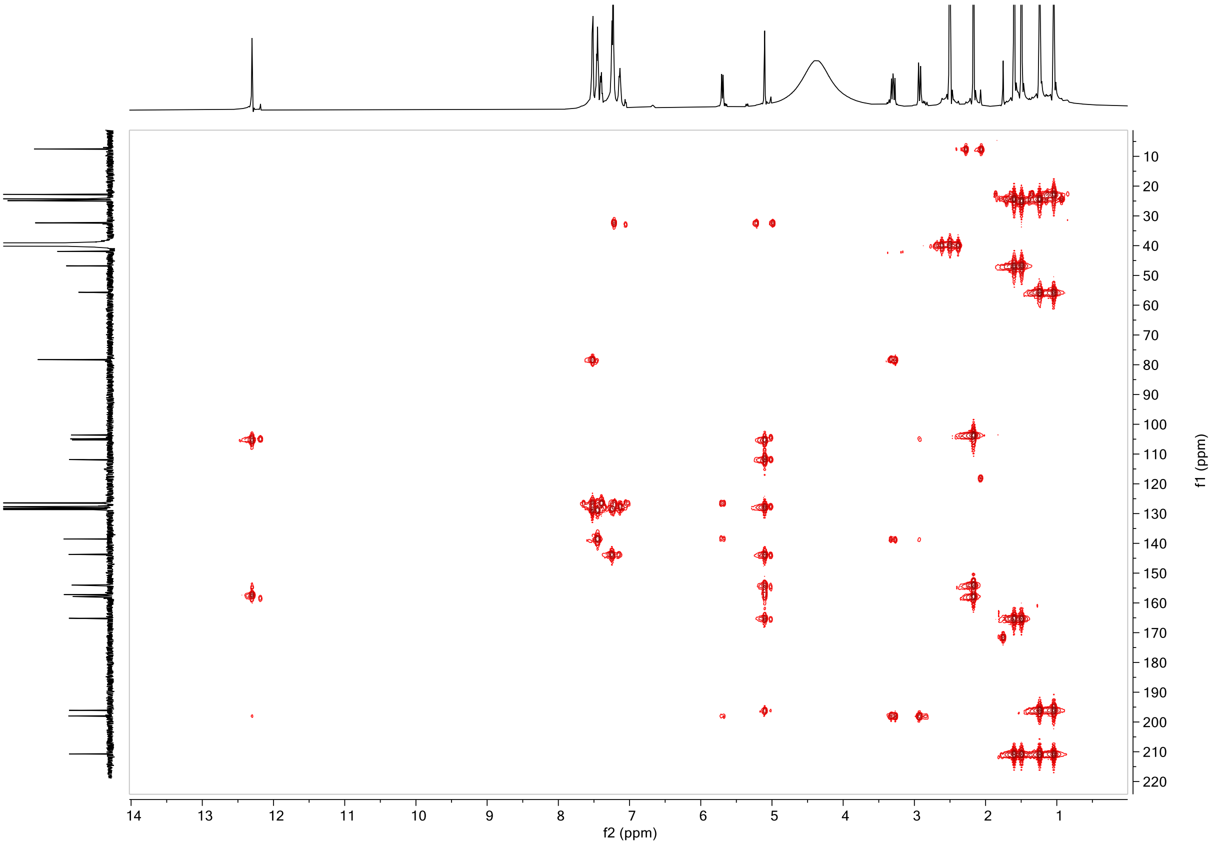

Figure S17. 1D ROESY Spectrum of **2** in DMSO-d_6 (irradiated at 5.70 ppm)

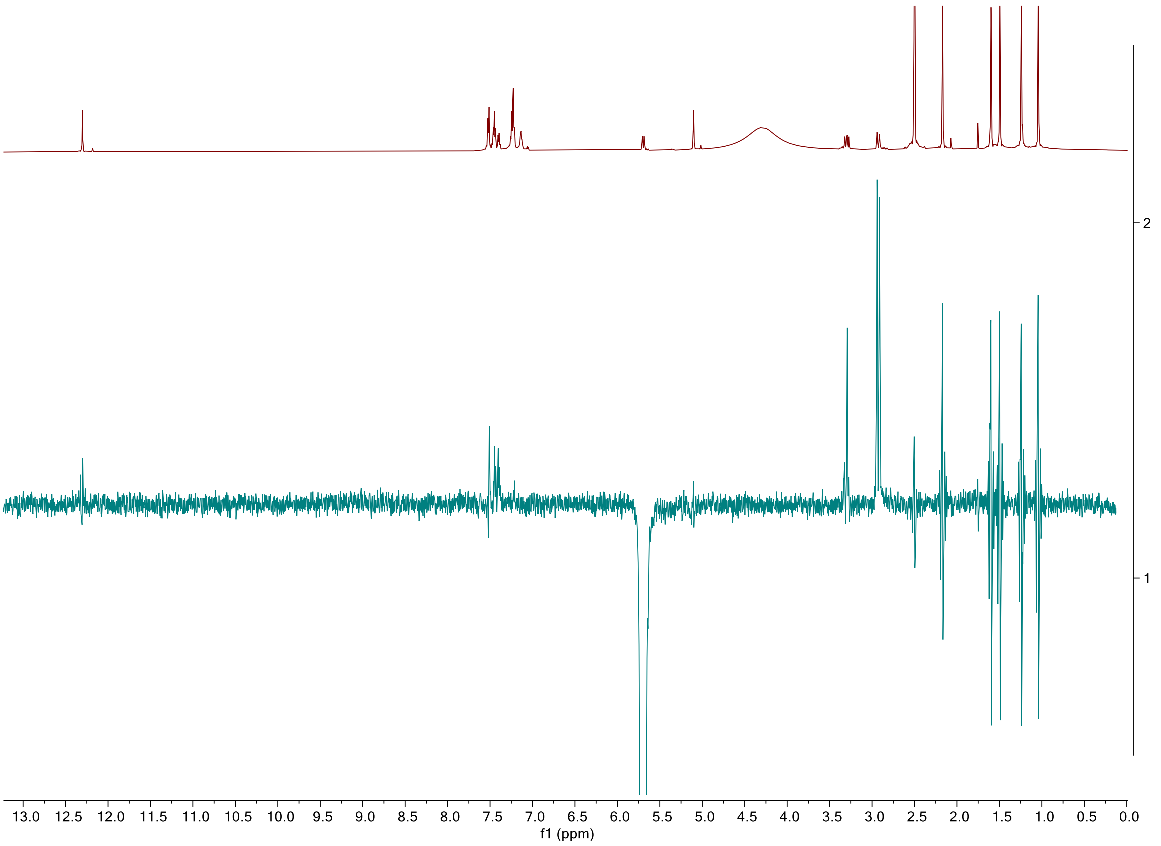

Figure S18. 1D ROESY Spectrum of **2** in DMSO-d_6 (irradiated at 5.10 ppm)

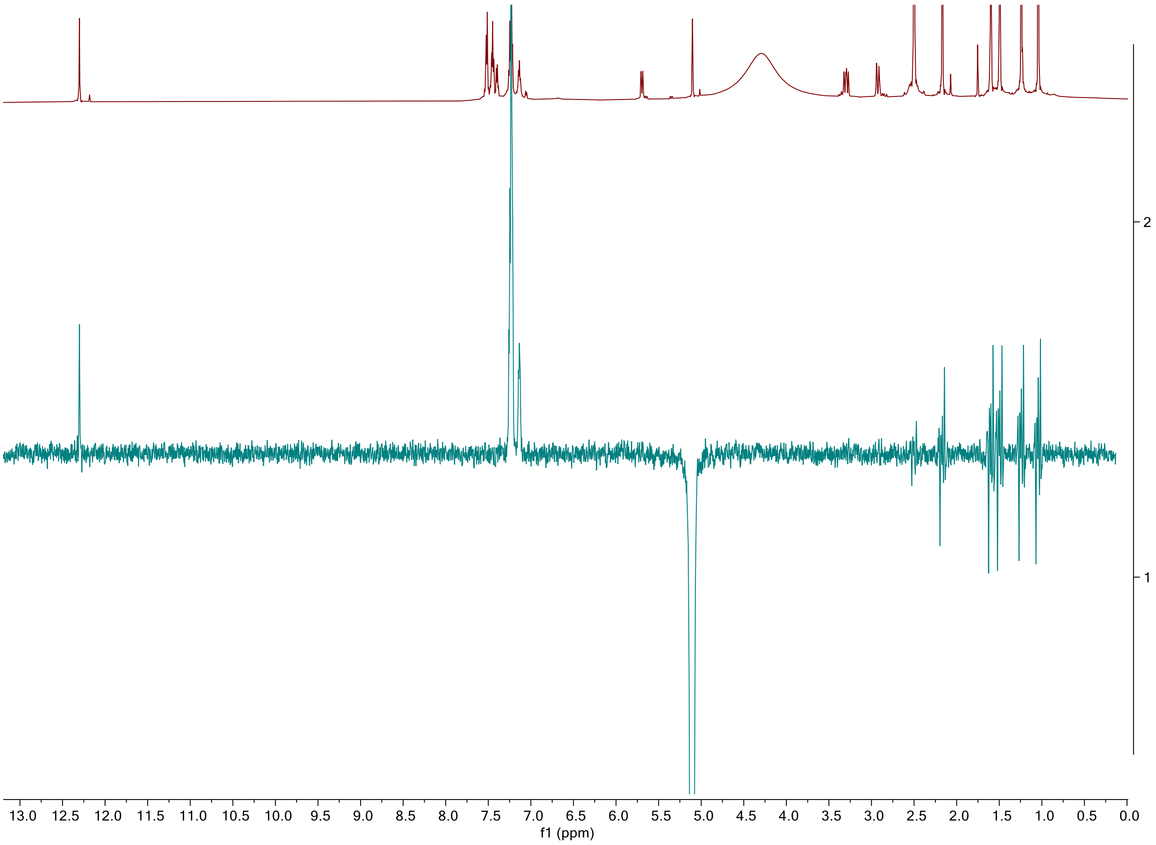

Figure S19. 1D ROESY Spectrum of **2** in DMSO-d_6 (irradiated at 2.17 ppm)

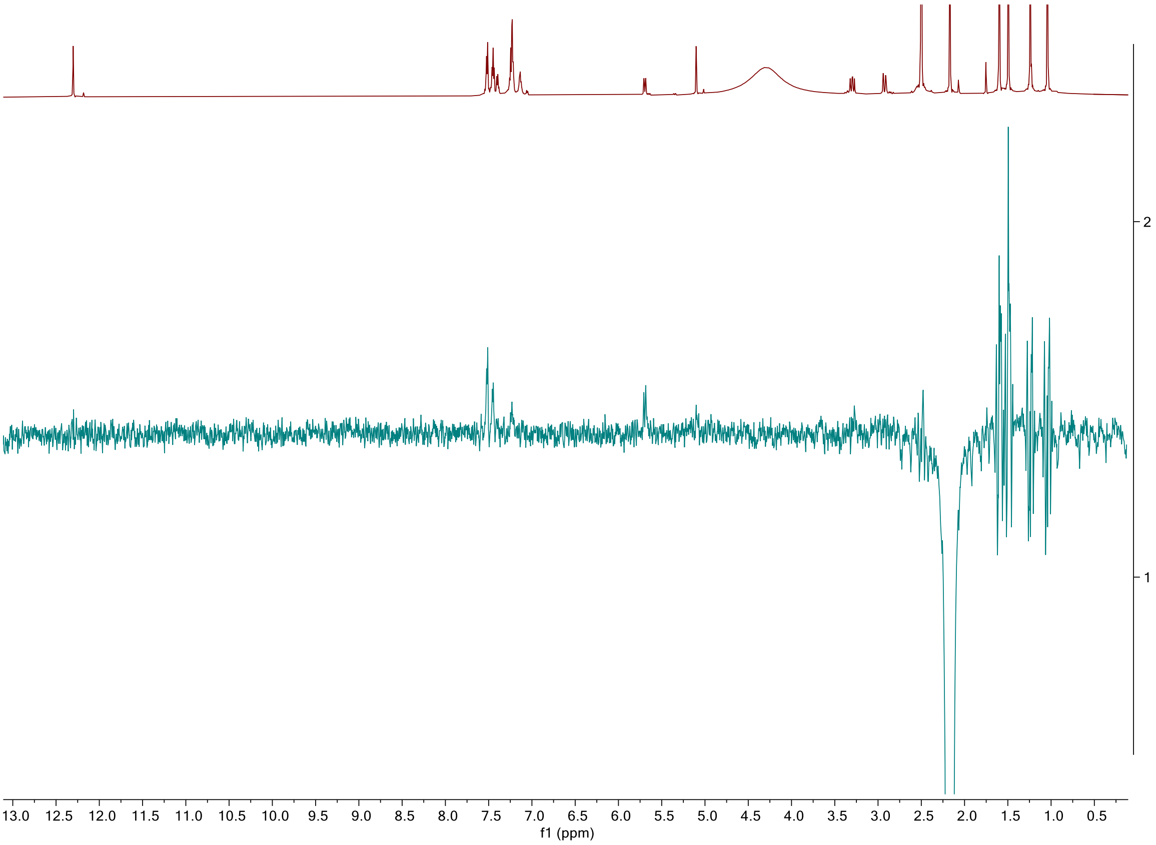

Figure S20. ^{1}H Spectrum of **2** in $CDCl_3$

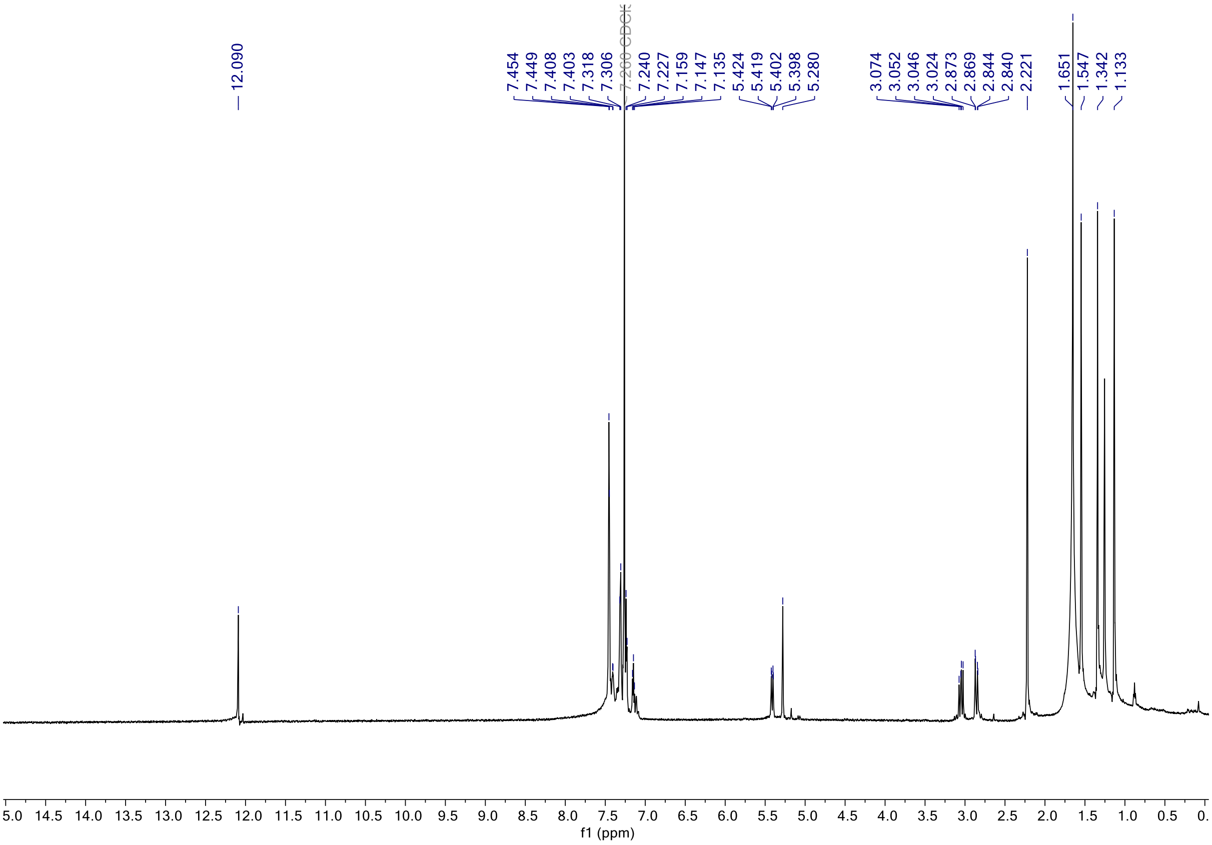

Figure S21. Cell viability assessed using the MTT assay after 24 h incubation of NFFs with 0.75-25 µM of **2** (A, n=8). NFF proliferation during 72 h treatment with **2** (B; **2**, n=2; DMSO, n=3). Data are mean±SEM.

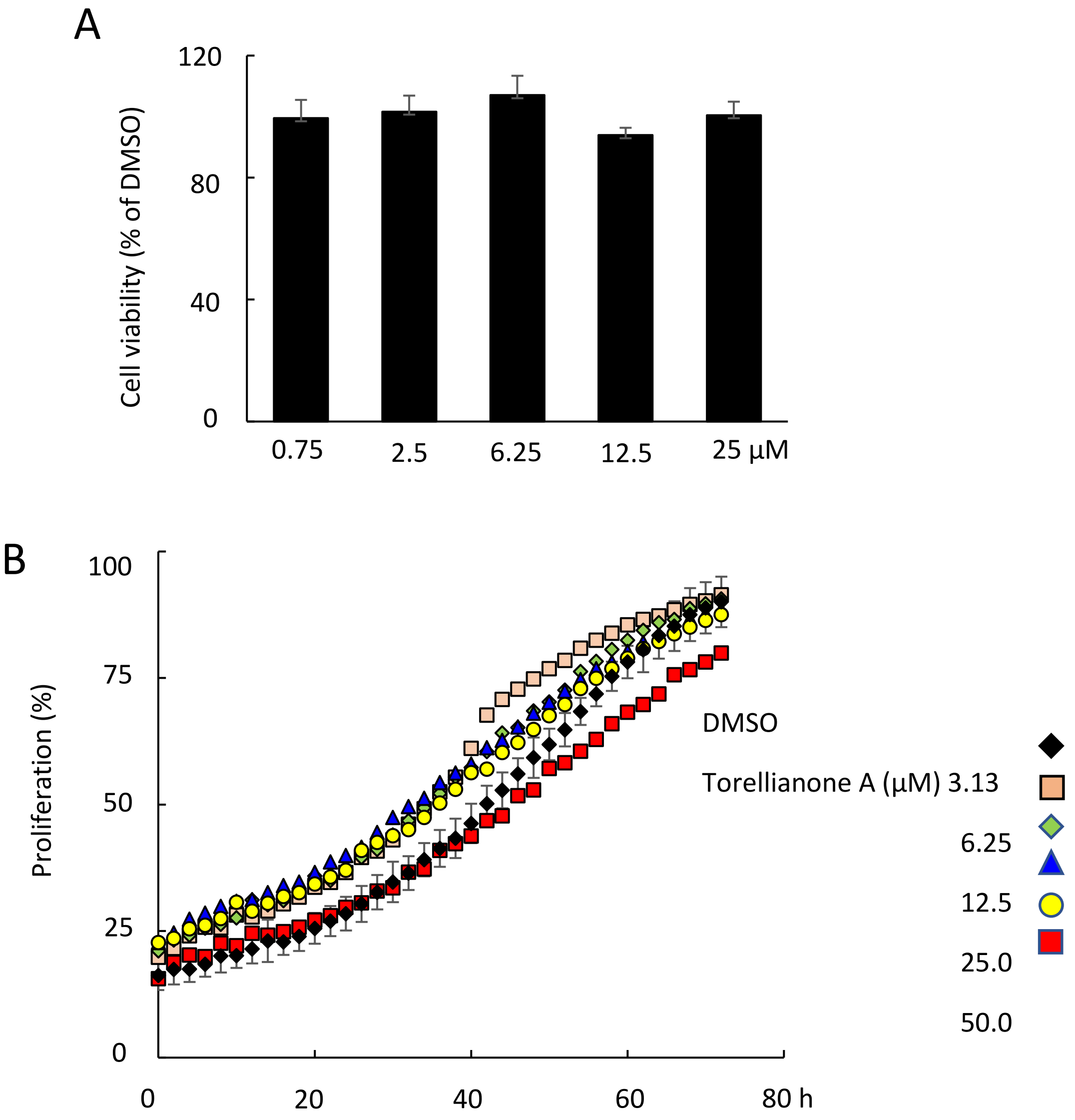

Figure S22. Effects of **2** (n=2) or DMSO (n=3) on wound repopulation by NFFs incubated for 72 h (n=2). Data was analysed for wound width (A) and rate of cell migration into the wound region (B).

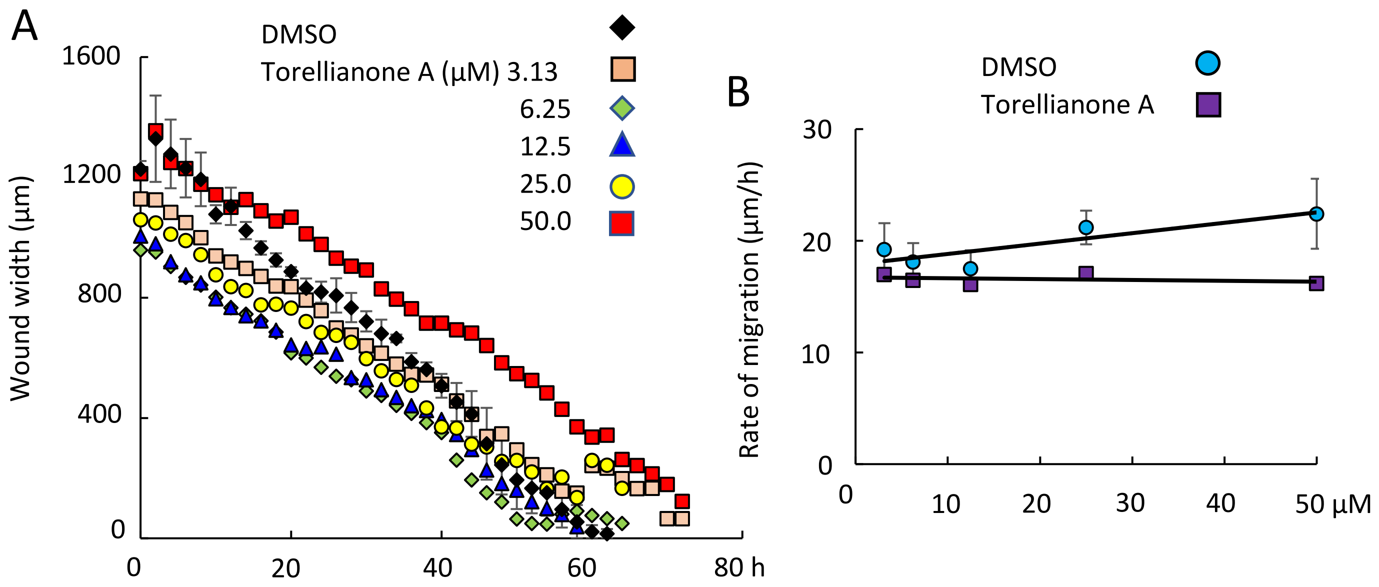

Supplement: Supplementary file 1 [file antioxidants-11-01604-s001.zip › antioxidants-1791858-supplementary.pdf]
